# The 6th International Leh Symposium

**DOI:** 10.1002/pul2.12109

**Published:** 2022-07-01

Leh, Ladakh, India 
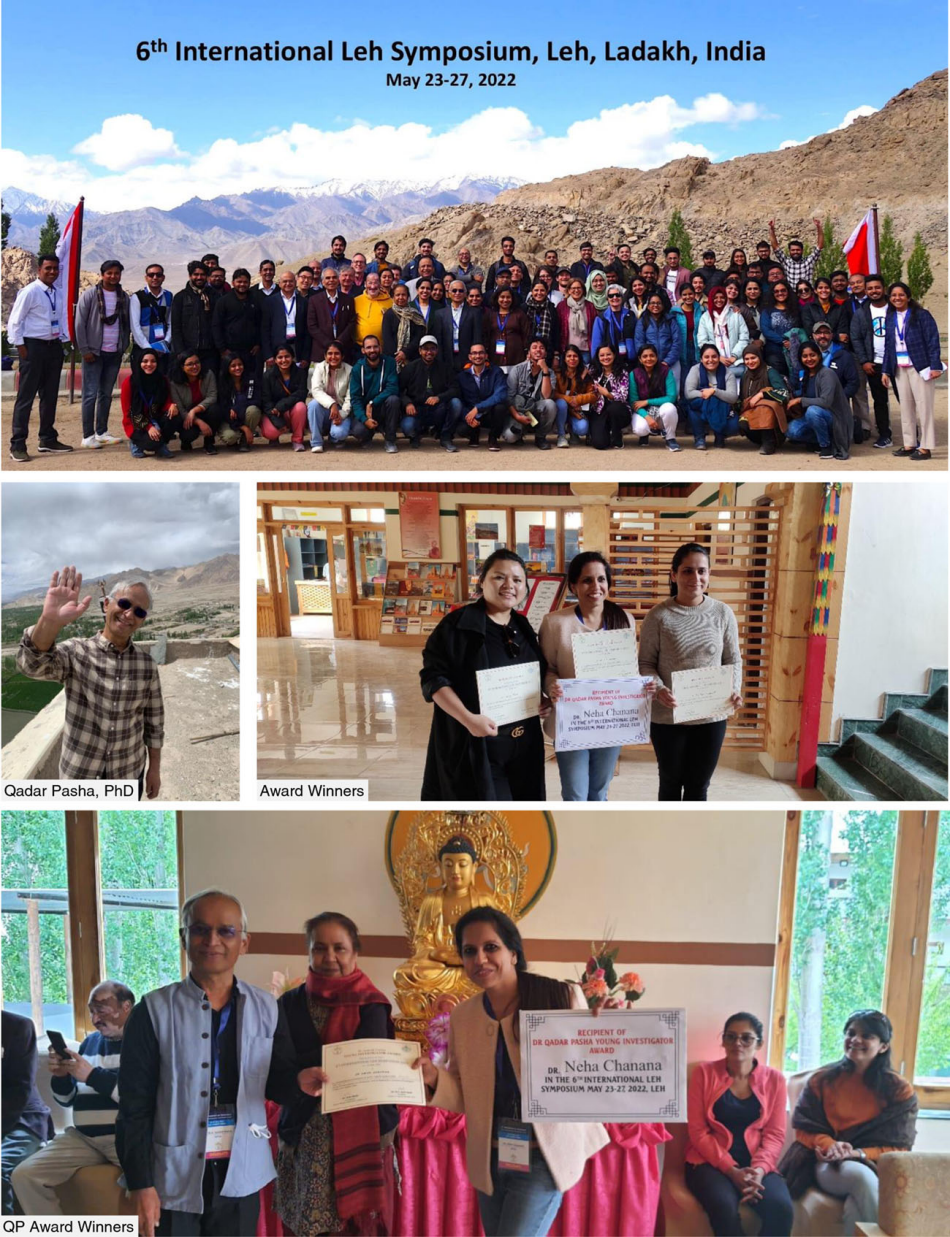


May 23–27, 2022

## ELIMINATING SENESCENT CELLS CAN PROMOTE PULMONARY HYPERTENSION DEVELOPMENT AND PROGRESSION

Serge Adnot

INSERM U955 and Département de Physiologie, Hôpital Henri Mondor, Université Paris‐Est Créteil (UPEC), Créteil, France

Senescent cells (SCs) are involved in proliferative disorders but their role in pulmonary hypertension (PH) remains undefined. We investigated SCs in patients with pulmonary arterial hypertension (PAH) and the role of SCs in animal PH models. We investigated senescence (p16, p21) and DNA damage (g‐H2AX, 53BP1) markers in patients with PAH and murine models. We monitored p16 activation by luminescence imaging in p16‐luciferase (p16^LUC/+^) knock‐in mice. SC clearance was obtained by a suicide gene (p16 promoter‐driven killer gene construct in p16‐ATTAC mice), senolytic drugs (ABT263 and cell‐permeable FOXO4‐p53 interfering peptide [FOXO4‐DRI]), and p16 inactivation in p16^LUC/LUC^ mice. We investigated PH in mice exposed to normoxia, chronic hypoxia, or hypoxia + Sugen; mice overexpressing the serotonin transporter (SM22‐5‐HTT^+^); and in rats given monocrotaline. Patients with PAH compared to controls exhibited high lung p16, p21, and g‐H2AX protein levels, with abundant vascular cells costained for p16, g‐H2AX, and 53BP1. Hypoxia increased thoracic bioluminescence in p16^LUC/+^ mice. In wild‐type mice, hypoxia increased lung levels of senescence and DNA‐damage markers, senescence‐associated secretory phenotype components, and p16 staining of pulmonary endothelial cells (P‐ECs, 30% of lung SCs in normoxia) and pulmonary artery smooth muscle cells. SC elimination by suicide gene or ABT263 increased the right ventricular systolic pressure and hypertrophy index, increased vessel remodeling (higher dividing proliferating cell nuclear antigen‐stained vascular cell counts during both normoxia and hypoxia), and markedly decreased lung P‐ECs. Pulmonary hemodynamic alterations and lung P‐EC loss occurred in aged p16^LUC/LUC^ mice, wild‐type mice exposed to Sugen or hypoxia + Sugen, and SM22‐5‐HTT^+^ mice given either ABT263 or FOXO4‐DRI, compared to relevant controls. The severity of monocrotaline‐induced PH in rats was decreased slightly by ABT263 for 1 week but was aggravated at 3 weeks, with loss of P‐ECs. Elimination of senescent P‐ECs by senolytic interventions may worsen pulmonary hemodynamics. These results invite consideration of the potential impact on pulmonary vessels of strategies aimed at controlling cell senescence in various contexts.

## ROLES OF CHROMATIN STRUCTURE, TRANSCRIPTION FACTORS, AND EPIGENETIC REGULATORS IN THE PERSISTENTLY ACTIVATED PHENOTYPE OF PULMONARY VASCULAR CELLS IN HYPOXIA‐INDUCED PULMONARY HYPERTENSION

Cheng J. Hu,^1,2^ Hui Zhang,^2^ Min Li,^2^ Kurt R. Stenmark^2^

^1^Department of Craniofacial Biology, School of DentaMedicine; University of Colorado Anschutz Medical Campus, Aurora, Colorado, USA; ^2^Cardiovascular Pulmonary Research Laboratories, Division of Pulmonary Sciences and Critical Care Medicine, Division of Pediatrics‐Critical Care, Departments of Medicine and Pediatrics, University of Colorado Anschutz Medical Campus, Aurora, Colorado, USA

Chronic hypoxia‐induced pulmonary hypertension (PH) is characterized by the accumulation of persistently activated cell types (endothelial cells, smooth muscle cells, and fibroblasts) in the pulmonary vessel. These cells exhibit increased expression of genes involved in inflammation, apoptosis resistance, proliferation, and metabolic reprogramming. Further, this persistently activated phenotype and aberrant gene expression in PH vascular cells are maintained in vitro, in the absence of complex in vivo environments, over multiple passages. Our hypothesis is that persistently overexpressed genes involved in inflammation (*CCL2*, *CSF2*, *IL‐6*, and *VCAM1*) in PH fibroblasts (PH‐Fib), the cells that are most predominate in the adventitia where inflammatory cells accumulate, are maintained epigenetically, due to an open/activated chromatin structure that is maintained by multiple transcription factors (TFs) and epigenetic regulators. Chromatin structure (using an assay for transposase‐accessible chromatin with high‐throughput sequencing [ATAC‐seq]) of genome‐wide genes was determined in control and PH‐Fibs from chronically hypoxic (15 days) bovine calves. ATAC‐seq revealed that a large number of genes (including cytokines and chemokines) whose expression is increased in bovine PH‐Fibs, exhibited significantly increased accessibility to transposase in PH‐Fibs, indicating “openness” in chromatin structure (promoters and/or enhancers), compared to the same set of genes in control‐Fibs. To determine the potential TFs that are involved in the expression of cytokines and chemokines in PH‐Fibs, the DNA sequence of the enhancers and/or promoters of cytokines/chemokines that were more open in PH‐Fibs were scanned for TF binding sites. We found that binding sites for TFs, including NF‐kb, STAT3, and AP1, were significantly enriched in the regulatory regions of cytokines/chemokines. The expression (by Western blots) and binding (by chromatin immunoprecipitation) of TFs and epigenetic regulators to inflammation genes in control and PH‐Fibs were determined. The levels of TFs including NF‐kb, AP1, and STAT3 and the epigenetic regulator BRD4 protein were significantly increased in PH fibs. Importantly, inflammation genes such as *CXCL12* and *CSF2* exhibited increased binding of these TFs and BRD4 in their promoters and/or enhancers in PH‐Fibs. We also examined the effect of targeting TFs or BRD4 on the expression of inflammation genes in PH‐Fibs. We found that inhibition of BRD4 binding, but not of single TFs, significantly reduced the expression of most inflammation genes in PH‐Fibs. Persistently overexpressed inflammatory genes in fibroblasts from the chronically hypoxic circulation are the result of open/activated chromatin structure that is maintained by multiple TFs and epigenetic regulators such as BRD proteins. Importantly, genes whose increased expression is maintained by one or more open enhancers that are bound with multiple TFs and BRD proteins can be effectively reversed by a BRD inhibitor but not by inhibitors targeting a single TF.

## TIBETAN ADAPTATION TO HIGH ALTITUDE: WHAT WE KNOW AND WHAT IS STILL MISSING?

Josef T. Prchal

Department of Internal Medicine, Division of Hematology and Hematologic Malignancies, University of Utah, Salt Lake City, Utah, USA

Tibetans have adapted for over ~25,000 years to their hypoxic homeland in the Himalayas. Hypoxia, via the modulation of levels of transcription factors known as hypoxia‐inducible factors (HIFs), regulates essential processes including erythropoiesis, blood vessel formation, and a unique energy metabolism of cancer cells known as the Warburg effect. There are two Tibetan‐specific evolutionary‐selected haplotypes, *EGLN1* (encoding for PHD2, the principal negative regulators of HIFs) and *EPAS1* (encoding for HIF‐2α). Both are crucial components of HIF pathways. The *EPAS1* haplotype introgressed from Denisovans and evolved with Denisovan and non‐Denisovan structures. We identified two nonsynonymous variants in the *EGLN1* gene (encoding PHD2), c.12C > G—unique to Tibetans, in complete linkage disequilibrium with c.380G > C, and showed it blunts hypoxic response. However, our analyses of 347 Tibetans living from 200 to 4500 m altitude found that while these *EGLN1* and *EPAS1* haplotypes provide some protection to the Tibetans from polycythemia, this protection is modest. *EGLN1* Tibetan haplotypes have also been shown to decrease exaggerated immune responses in hypoxia, likely accounting for Tibetan protection from acute mountain sickness; whether this haplotype also accounts for the Himalayan highlanders having recrudescence of tuberculosis when they move to low altitude remains to be shown. While it has long been argued that the Tibetan evolutionary adaptation to high‐altitude‐associated hypoxia is associated with lower‐than‐expected hemoglobin/hematocrit levels compared to low‐altitude dwellers (perhaps to protect them from high blood viscosity), it has also been reported that Tibetan Sherpas have a lower level of hemoglobin concentration than other sojourners to high altitude, but their expanded red cell volume is masked by a concomitant expansion of plasma volume. However, the large‐scale long‐read sequencing of Tibetan and Han samples reported that structural variations (SVs) are key drivers of selection under high‐altitude adaptation and may have contributed to even greater evolutionary selection than previously described haplotypes of *EPAS1* and *EGLN1*. We aim to determine the full functional impact of these selected haplotypes, SVs and copy number variations, and their relationship to measured red cell and plasma volumes and other Tibetan phenotypes.

## AGE DIFFERENCES IN CARDIOPULMONARY ADAPTATIONS TO HIGH ALTITUDE AND TIBETAN WOMEN'S REPRODUCTIVE SUCCESS

Cynthia M. Beall

Department of Anthropology, College of Arts and Sciences, Case Western Reserve University, Cleveland, Ohio, USA

High‐altitude human biology studies commonly focus on adults of reproductive age or children, representing the processes of acclimatization, developmental, and genetic adaptation. Postreproductive life merits attention because a loss of adaptive capacity characterizes biological aging, and parents' status can influence children's chances of survival and the next generation's gene pool. This report tests the hypothesis that lung volume and pulmonary blood pressure are associated with reproductive success among ethnic Tibetan women between 46 and 86 years of age who have completed their childbearing. Four hundred and seven native residents at 3500–4100 m in Upper Mustang, Nepal, provided anthropometry, pulmonary function, echocardiography, hypoxic ventilatory response, and reproductive history. General linear and analytic models selected influential covariates of reproductive success. Women with larger forced expiratory volume in six seconds and lower pulmonary artery systolic pressures had better reproductive success reflected by various measures, such as the number of live births and the number of children surviving to 15 years of age. This cross‐sectional study cannot distinguish between age differences and aging losses. Longitudinal studies of women of reproductive and postreproductive age will be required to detect the extent to which women with high reproductive success experience a slower loss of adaptive capacity or had a higher capacity during the reproductive years.

**Funding:** This research was supported by NSF awards 1153911 and 831530.

## ENDOTHELIAL PAS DOMAIN PROTEIN 1 VARIANTS REGULATE ADAPTATION AND MALADAPTATION MECHANISMS UNDER HYPOXIA

Tsering Palmo,^1,2^ Neha Chanana,^1,3^ Mohammed Faruq,^1^ Tashi Thinlas,^4^ Malik Z. Abdin,^2^  Qadar Pasha^1,5^

^1^CSIR‐Institute of Genomics and Integrative Biology, Delhi, India; ^2^Department of Biotechnology, Jamia Hamdard, New Delhi, India; ^3^Department of Biochemistry, Jamia Hamdard, New Delhi, India; ^4^Sonam N. Memorial Hospital, Leh, Ladakh, India; ^5^Institute of Hypoxia Research, New Delhi, India

Exposure to hypobaric hypoxic stress at high altitude (HA, >2500 m) induces several physiological processes that are regulated by hypoxia‐sensing genes in sojourners, who are susceptible to various HA disorders, such as high‐altitude pulmonary edema (HAPE). *Endothelial PAS domain 1* (*EPAS1*) is the master regulator of an oxygen‐sensing pathway at prolonged hypoxic exposure. The current study explored *EPAS1* gene in HA adaptation and maladaptation in three well‐characterized groups: 238 highland natives (HLs), 250 HAPE‐free controls (HAPE‐f), and 250 HAPE‐patients (HAPE‐p). Two single‐nucleotide polymorphisms (SNPs) of *EPAS1*, rs4953354 *A*/*G* and rs56721780 *G*/*C*, were genotyped in the three study groups. The protein levels of EPAS1 were determined from plasma and were correlated with each allele and genotype interactions. The relative expression of *EPAS1* was determined in peripheral blood leucocytes. The genotype distribution of rs4953354 *A*/*G* and *G*/*G* and rs56721780 *G*/*C* differed significantly in HLs when compared with HAPE‐f and HAPE‐p (*p* < 0.0001). The genotype rs4953354 *A*/*A*− rs56721780 *G*/*G* were overrepresented in HAPE‐p when compared with HLs (*p* < 0.0001). EPAS1 levels were significantly higher in HAPE‐p (*p* < 0.001 and 0.0001, respectively) and the gene expression complimented the bio‐level (*p* < 0.0001). The lower level of EPAS1 was significantly associated with elevated arterial oxygen saturation in HLs (*p* < 0.0001). The protective allele rs4953354 *G* was associated with decreased concentration of EPAS1 in HLs, whereas the reverse was seen with rs56721780 *C* (*p* < 0.001). Genotype interaction revealed overrepresentation of the risk–genotype combinations in HAPE‐p and protective‐allele‐associated genotype combinations in HLs. Allele‐specific transcriptional regulation of cellular *EPAS1* via dual‐luciferase assay, both under normoxic and hypoxic conditions, further confirmed their cellular functional role. In conclusion, the transcription and translation of *EPAS1* play a crucial role in the regulation of hypoxia‐inducible responses.

## HEMOGLOBIN INCREASE WITH ALTITUDE IN DIFFERENT WORLD REGIONS

Heimo Mairbäurl,^1,2^ Samuel Kilian,^3^ Martina U. Muckenthaler,^2,4^ Markus Thiersch,^5^ Rukundo K. Benedict,^6^ Max Gassmann^5,7^

^1^Translational Pneumology, University Hospital Heidelberg, Heidelberg, Germany; ^2^Translational Lung Research Center Heidelberg, Member of the German Center for Lung Research, Heidelberg, Germany; ^3^Institute of Medical Biometry and Informatics, University of Heidelberg, Heidelberg, Germany; ^4^Pediatric Oncology, Hematology and Immunology, University Hospital Heidelberg, Heidelberg, Germany; ^5^Institute of Veterinary Physiology, Vetsuisse Faculty and Zurich Center for Integrative Human Physiology, University of Zürich, Zürich, Switzerland; ^6^ICF, Demographic and Health Surveys, Rockville, Maryland, USA; ^7^Universidad Peruana Cayetano Heredia, Lima, Peru

Haemoglobin (Hb) values are elevated in high‐altitude residents. To define anaemia, the World Health Organization (WHO) uses ethnicity‐unspecific correction factors. However, empirical observations reveal that the WHO's correction overestimates the prevalence of anaemia but underestimates polycythaemia, particularly at altitudes over 3000 m. This is mainly caused by considerable variation in elevated Hb levels with an altitude between world regions with a maximal response observed in South Americans and a minimal in Tibetans. Hb values analyses performed by the Demographic and Health Surveys Program data sets confirm the divergent increase in the mean Hb values with an altitude between different world regions and suggest a nearly linear increase, rather than the previously suggested exponential increase. Results show for the first time that the increase in Hb concentration is lower in children <5 years of age compared to adults, and indicates that there is no consistent increase in some regions of Africa and in the Middle East; the reason for this remains yet unknown. The observed divergent alterations of the Hb values with altitude imply that a single algorithm cannot be applied worldwide for altitude adjustment of Hb concentrations. We propose region‐specific corrections that also account for age, sex, and pregnancy, and also additional research‐strengthening algorithms and tables to allow for a more accurate diagnosis of anaemia and polycythaemia in clinical practice.

## HYPOXIA CONDITIONING FOR PREACCLIMATIZATION, PROPHYLAXIS, AND THERAPY

Martin Burtscher

University of Innsbruck and the Austrian Society for Alpine and High‐Altitude Medicine, Innsbruck, Austria

Hypoxia conditioning (HC) has considerable potential to be applied (i) as an effective preacclimatization strategy before going to high altitudes, but may (ii) also be used to favorably modify the course of a variety of diseases. (1) In earlier times, exposure to hypobaric hypoxia (real altitude, hypobaric chamber) was the method of choice for preacclimatization. More than three decades ago, the application of normobaric hypoxia has been evaluated for this purpose by using a lightweight mobile device (the altitude‐conditioning apparatus). Subsequent findings from numerous studies performed in normobaric (but also hypobaric) hypoxia have led to the concepts of short‐ and long‐duration HC. For instance, participants of commercial expeditions spent (intermittently) about 300 h in hypoxia tents, reaching sleeping altitudes equivalent to 7100 m, before they rapidly and successfully ascended Mt. Everest (8849 m). (2) HC means that it is not hypoxia per se but adaptations initiated by hypoxia exposures, which may evoke health benefits. Distinct therapeutic and/or prophylactic effects from HC emerged from interventional studies using HC. Such calibrated HC programs improved exercise tolerance in healthy elderly individuals and in patients with cardiovascular or chronic pulmonary diseases, and also proved to be effective for the treatment and rehabilitation of patients with metabolic syndrome and those suffering from mild forms of neurodegenerative diseases. Nevertheless, the research field on HC is still in its infancy and will largely benefit from systematic evaluations regarding the effects of hypoxia parameters and the responsiveness of specified target groups.

## MEMBRANE AND CAPILLARY COMPONENT OF LUNG DIFFUSION CAPACITY IN HIGH‐ALTITUDE NATIVES

Vitalie Faoro

Cardio‐Pulmonary Exercise Laboratory, Faculty of Motor Sciences, Université Libre de Bruxelles, Bruxelles, Belgium

Several studies have shown that lung diffusion capacity at high altitude (HA), as measured by carbon monoxide transfer (DL_CO_), is higher in highlanders than in acclimatized lowlanders. However, major interstudy discrepancies have been reported, which may be attributed to methodological aspects (differences in techniques used), choice of the control group, ethnic differences, degree of hypoxia, and so on. Nevertheless, the higher DL_CO_ in the HA‐native and long‐term residents is substantial and persistent at exercise. Initially, it was thought that the increase in diffusion capacity was explained by larger lungs, with increased alveolar surface area and pulmonary capillary blood volume (Vc). According to the Roughton and Foster equation, the lung diffusion capacity is made up of two components, one due to the diffusion process across the alveolocapillary membrane (Dm) and one attributable to the reaction of the tracer gas with hemoglobin (qVc). Double tracer gas (CO and NO) or double oxygen tension techniques allow for Dm and Vc calculation. Both components have been shown to be increased in highlanders at HA. Interestingly, this observation persists even after correction for the alveolar volume and the increase in Vc is disproportionate to that of Dm. How much distension and recruitment of capillaries are involved in increasing Vc remains unknown, but a hypoxia‐induced angiogenesis is suspected. Additional studies using biological markers are needed to confirm the existence of an angiogenic process. It also needs to be clarified to what extent this adaptation may benefit pulmonary hemodynamics by decreasing the resistance to blood flow and benefits gas exchange with a subsequent decreased ventilatory response at exercise.

## CIRCULATING EXTRACELLULAR VESICLES CARRYING THROMBOINFLAMMATORY PROTEINS PROMOTE PULMONARY VASCULAR INJURY IN COVID‐19 PATIENTS

Balaji Krishnamachary,^1^ Christine Cook,^1^ Leslie Spikes,^1^ Prabhakar Chalise,^2^ Navneet K. Dhillon^1^

^1^Division of Pulmonary and Critical Care Medicine, Department of Internal Medicine, University of Kansas Medical Center, Kansas City, Kansas, USA; ^2^Department of Biostatistics and Data Science, University of Kansas Medical Center, Kansas City, Kansas, USA

It is increasingly recognized that moderate/severe coronavirus disease 2019 (COVID‐19) disease is in part due to a dysregulated immune response in conjunction with increased thromboinflammation, coagulopathy, and vascular injury. In this study, we analyzed the cargo of extracellular vesicles (EVs) isolated from the plasma of patients with COVID‐19 for the identification of potential biomarkers of disease severity and to explore their role in disease pathogenesis. Severe acute respiratory syndrome coronavirus 2‐infected patients hospitalized at the University of Kansas Health System were enrolled in the COVID‐19 In‐patient Biorepository and blood samples were collected for the isolation of plasma‐derived EVs. The patients were grouped based on the WHO Clinical Progression Scale score during hospitalization. Our results revealed enrichment of proinflammatory, procoagulation, and tissue‐remodeling markers in circulating EVs, distinguishing symptomatic COVID‐19 patients from uninfected controls and delineating patients with moderate disease from the critically ill. Among all proteins analyzed, the levels of proinflammatory DAMP: EN‐RAGE (aka calgranulin C or S100A12) showed the strongest correlation with length of hospitalization and disease severity. In addition, tissue factor levels/activity linked to EVs appeared to distinguish patients with severe disease from those with a moderate disease but on supplemental oxygen. Alterations in EV cargo corresponded to enhanced apoptosis of primary human pulmonary microvascular endothelial cells and smooth muscle hyperplasia on exposure to EVs from COVID‐19 patients. In conclusion, our findings suggest a pivotal role of EVs in the pathogenesis of acute COVID‐19 disease. Whether these EV‐mediated alterations continue leading to long‐haul COVID including cardiopulmonary vascular complications is the focus of our ongoing studies.

## COVID‐19 WITH RESPECT TO PROTECTION OR RISK AT HIGH ALTITUDE: A GLOBAL PERSPECTIVE

Bilal Ahmed Abbasi,^1^ Neha Chanana,^2^ Tsering Palmo,^1,^ ^2^ Qadar Pasha^1,2^

^1^CSIR‐Institute of Genomics and Integrative Biology, Delhi, India; ^2^Institute of Hypoxia Research, New Delhi, India

Severe acute respiratory syndrome coronavirus 2 (SARS‐CoV‐2) has affected every demography disproportionately, including even the native highland populations. Hypobaric–hypoxic settings at high altitudeS (HAS, >2500 m) present an extreme environment that impacts the survival of permanent residents, possibly including SARS‐CoV‐2. Conflicting hypotheses have been presented for coronavirus disease 2019 (COVID‐19) incidence and fatality at HA. To evaluate protection or risk against COVID‐19 incidence and fatality in humans under hypobaric–hypoxic environment of HA (>2501 masl). Global COVID‐19 data for March 2020–2021 obtained from official websites of the Indian Government, John Hopkins University, and Worldometer were clustered into six altitude categories. Clinical cofactors and comorbidities data were evaluated with COVID‐19 incidence and fatality. Extensive comparisons and correlations using several statistical tools estimated the risk and protection. Of relevance, data analyses revealed four distinct responses, namely, partial risk, total risk, partial protection, and total protection from COVID‐19 at HA, indicating a mixed baggage and complexity of the infection. Surprisingly, it included the countries within the same geographic region. Moreover, body mass index, hypertension, and diabetes correlated significantly with COVID‐19 incidence and fatality rate (*p* ≤ 0.05). Varied patterns of protection and risk against COVID‐19 incidence and fatality were observed among the HA populations. However, it is premature to generalize COVID‐19 effects on any particular demography without further extensive studies.

## HIGH‐ALTITUDE PULMONARY EDEMA PRECIPITATED BY SARS‐COV‐2 INFECTION: A CASE PRESENTATION

Stanzen Rabyang

Sonam Norboo Memorial Hospital, Leh, Ladakh, India

Ladakh is a high‐altitude area of India, with its altitude ranging from 2500 to 6000 m, and it has been one of the famous tourist destinations for explorers, trekkers, and mountaineers. Every year a large number of tourists, as well as laborers, visit Ladakh, risking their lives to high‐altitude illnesses ranging from acute mountain sickness to fulminant high‐altitude cerebral edema and high‐altitude pulmonary edema (HAPE). HAPE is a life‐threatening altitude illness that usually occurs in insufficiently acclimatized climbers in the first few days at altitudes >2500 m. Apart from genetic susceptibility, mode, and rate of ascent, upper airway infections pose a risk determinant of HAPE. The hallmark of HAPE is hypoxic pulmonary vasoconstriction. Symptoms of HAPE are incapacitating fatigue, dyspnea at a minimal effort that advances to dyspnea at rest, and dry cough progressing to productive cough expectorating pinkish frothy sputum. The main modalities of treatment are immediate descent to lower altitude areas, hyperbaric oxygen therapy, and pulmonary vasodilator. For prophylaxis, slow ascent at the rate of 300 m/day and acetazolamide 500 mg/day 2 days before ascent is recommended. The objective of this report is to know the diagnostic and therapeutic approach of HAPE since it is confused with other pathologies presenting with respiratory failure. Little is known about viral infections as a risk factor for HAPE. Emphasis has been given to severe acute respiratory syndrome coronavirus 2 (SARS‐CoV‐2) infection as a risk determinant for HAPE, which is a rare case. To date, there is no study on SAR‐CoV‐2 as a risk determinant of HAPE. We report here a case of a 35‐year‐old young male, laborer from Bihar (India) with previous high‐altitude experience ascending to Leh Ladakh on July 16, 2020 by flight. He got admitted to SNM Hospital Leh on  July 24, 2020 with complaints of fever, body aches, and pain 2 days before ascending. After 3 days at altitude, he also developed a cough, which was initially dry but within a few days became productive with expectoration of pink frothy sputum. He also developed breathlessness on exertion, progressing to severity at rest within few days. There was no history of chest pain, orthopnea, and paroxysmal nocturnal dyspnea. General physical examination revealed Glasgow Coma Scale of 15/15, sick looking, respiratory rate of 24 breaths/min, oxygen saturation (SpO_2_) of 65% in room air, blood pressure of 110/70 mmHg, pulse rate of 120 b/min regular, and temperature of 99.8 F. Chest examination showed bilateral coarse crackles on auscultation. The rest of the systemic examinations were unremarkable. Chest radiograph revealed bilateral patchy nonhomogenous alveolar opacities more on the right side with hilar haze. A provisional diagnosis of HAPE was made with the differential diagnosis of coronavirus disease 2019 (Covid‐19) pneumonia. He was managed on the line of HAPE. The next day, his nasopharyngeal swab for Covid‐19 reverse transcription‐PCR (RT‐PCR) test turned out to be positive, but his general condition had markedly improved maintaining a SpO_2_ of 93% on 2–3 L/min and RR of 18/min. Repeat chest radiograph on Day 4 was within normal. He got discharged from the hospital on Day 22 of admission after a nasopharyngeal swab for RT‐PCR turned out to be negative.

## MANAGING PULMONARY ARTERIAL HYPERTENSION IN A RESOURCE‐DIVERSE COUNTRY LIKE INDIA

Prashant Bobhate

Children Heart Centre, Kokilaben Dhirubhai Ambani Hospital, Mumbai, India

Limited data on the etiology, demography, and short‐term outcome of patients with pulmonary arterial hypertension (Group 1 PAH) is available from low‐ and middle‐income countries, including India. We conducted a retrospective study of 523 patients with pulmonary hypertension, from a quaternary referral center in Western India. Pulmonary hypertension was mainly diagnosed based on either cardiac catheterization (patients without congenital heart disease) or Doppler echocardiogram (patients with congenital heart disease). The primary outcome was a composite endpoint of all‐cause mortality, use of advanced pulmonary vasodilators (prostacyclin analogs), listing for a lung transplant, and performing a reverse Potts shunt. The mean age of the study population was 56 ± 16 years. The most common cause of PAH was congenital heart disease (39%), followed by idiopathic or heritable PAH (32%), connective tissue disease 23%, and miscellaneous (6%). Nearly two‐thirds (65%) of the study participants had functional Class II symptoms at the time of enrollment. The median follow‐up was 17 months (3.2–83 months). Of the patients, 71% were on specific pulmonary vasodilators, with a majority (53%) receiving dual combination therapy. In total, 86 patients died during the follow‐up period, 29 patients were initiated on prostacyclin analogs, 22 patients underwent reversed Potts's shunt, and four patients were listed for lung/heart–lung transplant. In the multivariate model, baseline NYHA functional Class III/IV (odds ratio [OR]: 1.85, 95% confidence interval [CI]: 1.35–3.23), younger age at diagnosis (OR: 0.85, 95% CI: 0.78–0.94) and absence of intra‐ or extracardiac shunt (OR: 1.80, 95% CI: 1.29–2.51) were associated with primary composite outcome (*p* < 0.05). In our study, the mortality rate was 16.4%. Younger age and worse functional class at diagnosis and absence of shunt lesion were associated with worse clinical outcomes. Judicious use of reversed Potts's shunt and prostacyclin analogs reduced the mortality in patients without shunt lesions.

## A CONTROL OF OXIDATIVE/NITROSATIVE STRESSES MITIGATES DOXORUBICIN‐INDUCED CARDIOMYOPATHY

Pawan K. Singal

Institute of Cardiovascular Sciences, St. Boniface Hospital Albrechtsen Research Centre, Department of Physiology and Pathophysiology, Rady Faculty of Health Sciences, University of Manitoba, Winnipeg, Manitoba, Canada

We have earlier reported that an increase in oxidative stress is the main cause of doxorubicin (Dox)‐induced cardiotoxicity. However, there is now evidence that activation of inducible nitric oxide synthase (iNOS) and nitrosative stress are also involved. The present study, using isolated cardiomyocytes, investigated the effects of vitamin C (Vit C) in the mitigation of Dox‐induced changes in the levels of nitric oxide (NO), NOS activity, protein expression of NOS isoforms, and nitrosative stress. Cytokines tumor necrosis factor‐α (TNF‐α) and interleukin (IL‐10) in these isolated cardiomyocytes were also analyzed. Dox caused a significant increase in the generation of superoxide radical (O_2_^−.^), peroxynitrite, and NO, and these effects of Dox were blunted by Vit C. Dox increased the expression of iNOS and altered protein expression as well as activation of endothelial NOS (eNOS). These changes were prevented by Vit C. Dox induced an increase in the ratio of monomeric/dimeric eNOS, promoting the production of O_2_^−.^, which was prevented by Vit C by increasing the stability of the dimeric form of eNOS. Vit C protected against Dox‐induced increase in TNF‐α as well as a reduction in IL‐10. These data suggest that Vit C provides cardioprotection by reducing oxidative/nitrosative stress and inflammation by modulating Dox‐induced increase in the NO levels and NOS activity.

**Funding:** This study was supported by the Heart and Stroke Foundation of Canada.

## STEM CELL‐ AND BIOMATERIAL‐BASED REGENERATIVE STRATEGIES FOR HYPOXIC HEART

Sanjiv Dhingra

Institute of Cardiovascular Sciences, St. Boniface Hospital Research Centre, Regenerative Medicine Program, Department of Physiology and Pathophysiology, Rady Faculty of Health Sciences, University of Manitoba, Winnipeg, Manitoba, Canada

Bone marrow‐derived mesenchymal stem cells (MSCs) and induced pluripotent stem cell‐derived cardiomyocytes are being touted as ideal options for cardiac regeneration and repair after myocardial infarction. The outcome of experimental animal studies and initial clinical trials was positive and there were no side effects observed after the transplantation of stem cells. In fact, the implanted cells were able to improve heart function. However, the poor survival of transplanted cells in the recipient's heart has significantly prevented the translation of allogeneic (donor‐derived) stem cell‐based therapies to the clinic. In our recent findings, we have demonstrated that after transplantation in the hypoxic/ischemic heart allogeneic stem cells become immunogenic and are rejected by the host immune system. Therefore, we became interested in developing strategies to prevent immune rejection and improve the survival of transplanted stem cells in the ischemic heart. We synthesized and characterized 0D titanium carbide MXene quantum dots (MQDs). Our recent data report that MQDs possess intrinsic immunomodulatory properties and selectively reduce activation of human CD4^+^IFN‐γ^+^ T lymphocytes and promote the expansion of immunosuppressive CD4^+^CD25^+^FoxP3^+^ regulatory T cells in an activated lymphocyte population. The MQDs are biocompatible with stem cells. In our studies, we also incorporated MQDs into a chitosan‐based hydrogel to create a 3D platform for stem cell delivery to the heart. This composite immunomodulatory hydrogel‐based platform improved the survival of stem cells and mitigated alloimmune responses. These studies highlight the potential of MXene‐based next‐generation biomaterials for cardiac tissue engineering and stem cell‐based therapies for cardiac regeneration.

## MYOCARDIAL INFARCTION‐INDUCED HYPOXIA REGULATES CARDIAC LYMPHANGIOGENESIS

Kumaravelu Jagavelu

Pharmacology Division, CSIR‐Central Drug Research Institute, Lucknow, Uttar Pradesh, India

Myocardial infarction (MI) causes massive cardiomyocyte loss and replacement by noncontractile scar tissue, leading to pathological remodeling and eventually heart failure. Lymphatic performs a crucial role in the removal of fluid and immune cell tissue; therefore, cardiac lymphangiogenesis has recently been recommended as a therapeutic target for preventing heart failure. MAPKAPK2 (MK2) plays a crucial role in a variety of physiological functions, including stress and inflammatory responses. Here, we studied the effect of MK2 on lymphangiogenesis during MI. Echocardiographic and histological analysis showed MK2 inhibition improved cardiac functional characteristics after MI. MK2 deficiency led to the increased expression of lymphatic markers such as LYVE1, PROX1, VEGFC, and VEGFR3 and inflammation. Lymphangiography showed that the MK2/KO mice have more lymphatic neovascularization post‐MI. In vitro investigations with human dermal lymphatic cells promote lymphangiogenesis in MK2‐silenced cells after hypoxia. In conclusion, MK2 deletion improves cardiac lymphatic vasculature, with reduced inflammation, thus resulting in reduced MI‐induced damage.

## OPTIONS FOR NEUROPROTECTION FROM HYPOXIC BRAIN INJURY AFTER CARDIAC ARREST?

Olga M. Wagner

Charité—Universitätsmedizin Berlin, Corporate Member of Freie Universität Berlin and Humboldt‐ Universität zu Berlin, Berlin, Germany

In recent years, options for neuroprotection in the intensive care unit (ICU) in postcardiac arrest patients have been discussed and established. Volatile anesthetics have been proven to be a feasible and safe means of sedation in intensive care patients in postresuscitation treatment. Basic research and animal studies have shown promising neuroprotective effects from hypoxic brain injury. In an observational propensity‐score matched study, we looked at the neurological outcome in 432 patients undergoing postcardiac arrest treatment in the ICU of Charité University Hospital Berlin. While 322 patients received intravenous sedation, 110 patients were treated with volatile anesthesia (isoflurane via anesthetic‐conserving device). Although feasibility and safety of volatile anesthetic agents (isoflurane) in ICU treatment after cardiac arrest with a significantly shorter time on ventilator and ICU stay could be confirmed, cerebral performance category (CPC) scores of patients did not differ at hospital discharge (CPC 1–2 of 45% in both groups). In postcardiac arrest patients treated with volatile sedation, neuroprotective effects manifested as the improved neurological outcomes could not be shown. We are currently evaluating newer data extracted from a similar patient collective in which a new anaesthetic‐conserving device with reduced dead space ventilation was used. The new device was introduced to reduce hypercapnia, which was a major adverse event leading to the termination of volatile sedation in previous studies. Furthermore, prospective, controlled trials are needed to confirm these findings.

## BONE MORPHOGENIC PROTEIN RECEPTOR 2 MUTATIONS IN SOUTH INDIAN POPULATION WITH PULMONARY ARTERIAL HYPERTENSION

Shine Kumar,^1^ Lalitha Biswas,^2^ Chethampadi Gopi Mohan,^3^ Raman Krishna Kumar^4^

^1^Department of Pediatric Cardiology, Pulmonary Hypertension Clinic, Amrita Institute of Medical Sciences, Amrita Vishwa Vidyapeetham University, Kochi, Kerala, India; ^2^Centre for Nanosciences and Molecular Medicine, Amrita Institute of Medical Sciences, Amrita Vishwa Vidyapeetham University, Kochi, Kerala, India; ^3^Centre for Nanosciences and Molecular Medicine, Amrita Institute of Medical Sciences, Amrita Vishwa Vidyapeetham University, Kochi, Kerala, India; ^4^Department of Pediatric Cardiology, Amrita Institute of Medical Sciences, Amrita Vishwa Vidyapeetham University, Kochi, Kerala, India

Bone morphogenic protein receptor 2 (BMPR2) mutations are described in patients with pulmonary arterial hypertension (PAH). We sought to describe the characteristics of BMPR2 mutations in a selected population from South India. All patients with a diagnosis of idiopathic PAH (IPAH), hereditary PAH (HPAH), and PAH disproportionate to congenital heart disease (CHD) were included. BMPR2 mutation was analysed in the peripheral vein blood sample by the Sanger sequencing method. The study period was from January 2018 to October 2019. We identified nine BMPR2 mutations in 15 patients (including two carriers) among 52 (28.8%) analysed, with the majority being females (10, 66.6%). The median age at diagnosis was 22 years (range: 0.7–52 years). The diagnosis was IPAH (60%), HPAH (33.3%), and PAH with associated CHD (6.6%). All were heterozygous with missense (55.5%), nonsense (22.2%), frameshift (11.1%), and 3′‐untranslated region (UTR) (11.1%) mutations. Mutations were found in exons 3, 7, 9, 11, and 12 and in 3′‐UTR regions of the BMPR2 gene. There were two novel mutations. The HPAH patients had mutation with autosomal dominant inheritance and variable penetrance. All patients had severe PAH at diagnosis with suboptimal therapeutic response. The novel HPAH mutation had an excellent response to imatinib. After a mean follow‐up period of 43.7 ± 21.3 months, there was two mortality (13.3%). In the South Indian population, BMPR2 mutations have variable age at onset, with female preponderance and severe PAH at diagnosis. Overall prognosis seems guarded with a unique response to imatinib in a novel HPAH mutant.

## POTENTIAL PATHOGENETIC CONTRIBUTION OF HIF1Α‐RELATED GLYCOLYTIC ENZYMES IN LUNG INTERSTITIAL MACROPHAGES TO *SCHISTOSOMA*‐INDUCED PULMONARY HYPERTENSION

Michael H. Lee,^1^ Claudia Mickael,^2^ Linda Sanders,^2^ Biruk Kassa,^1^ Rahul Kumar,^1^ Brian B. Graham^1^

^1^Department of Medicine, Division of Pulmonary and Critical Care Medicine, University of California San Francisco, San Francisco, California, USA; ^2^Department of Medicine, Division of Pulmonary Sciences and Critical Care Medicine, University of Colorado, Aurora, Colorado, USA

Rodent studies of experimental pulmonary hypertension (PH) have demonstrated the protective roles of inhibiting hypoxia‐inducible factor‐1α (HIF1α) and its associated glycolytic genes in endothelial and smooth muscle cells. However, despite increased glucose uptake by the whole lungs, precise pathobiological relevance of glycolysis in the two vascular layers has not been established in humans beyond cell‐based studies, suggesting the potential contribution of HIF1α‐induced glycolysis in perivascular immune cells. We, therefore, interrogated HIF1α‐associated glycolytic gene expression in lung interstitial macrophages (IMs) of mice during the course of *Schistosoma*‐PH development. Four male and female pairs of C–X3–C motif chemokine receptor 1‐green fluorescent protein reporter mice, in which monocytes and IMs express GFP, were intraperitoneally sensitized with *Schistosoma mansoni* eggs. After 2 weeks, three pairs were also challenged with intravenous (IV) *S. mansoni* eggs, and their lungs were harvested 1, 3, or 7 days later. One pair of mice that did not receive IV eggs served as controls. GFP+, CD64+, CD11b^high^, and CD11c^intermediate^ IMs were isolated with fluorescence‐activated cell sorting, and single‐cell RNA sequencing was performed. Compared to the control mice, we observed upregulated transcription of HIF1α and most of its glycolytic targets at all three times points, including hexokinase, 6‐phosphofructokinase, platelet type, fructose‐bisphosphate aldolase A, triosephosphate isomerase, glyceraldehyde‐3‐phosphate dehydrogenase, phosphoglycerate kinase 1, phosphoglycerate mutase 1, α‐enolase, and pyruvate kinase. Statistical overrepresentation tests compared to the PANTHER and Reactome databases confirmed significant upregulation of the glycolysis pathway in the lung IMs. It may be concluded that transcription of HIF1α and its glycolytic targets is upregulated in lung IMs during murine *Schistosoma*‐PH development. The pathobiological importance of this finding requires further validation, including confirmation of HIF1α protein stabilization and quantification of glycolysis in the lung IMs.

## SEXUAL DIMORPHISM OF DEXAMETHASONE AS A PROPHYLACTIC TREATMENT IN PATHOLOGIES ASSOCIATED WITH ACUTE HYPOBARIC HYPOXIA EXPOSURE

Neha Chanana,^1,2^ Tsering Palmo,^1^ Kavita Sharma,^2^ Rahul Kumar,^3^ Bhushan Shah,^4^ Sudhanshu Mahajan,^4^ Girish M. Palleda,^4^ Mohit D. Gupta,^4^ Ritushree Kukreti,^1^ Mohammad Faruq,^1^ Tashi Thinlas,^5^ Brian B. Graham,^3^ Qadar Pasha^1,6^

^1^Department of Genomics and Molecular Medicine, CSIR‐Institute of Genomics and Integrative Biology, Delhi, India; ^2^Department of Biochemistry, Jamia Hamdard University, New Delhi, India; ^3^Department of Medicine, University of California San Francisco, San Francisco, California, USA; ^4^Department of Cardiology, GB Pant Institute of Post Graduate Medical Education and Research, New Delhi, India; ^5^Department of Medicine, Sonam Norboo Memorial Hospital, Leh, Ladakh, India; ^6^Institute of Hypoxia Research, New Delhi, India

Dexamethasone can be taken prophylactically to prevent hypobaric hypoxia‐associated disorders of high altitude. While dexamethasone‐mediated protection against high‐altitude disorders has been clinically evaluated, detailed sex‐based mechanistic insights have not been explored. As part of our India‐Leh‐Dexamethasone‐expedition‐2020 (INDEX2020) program, we examined the phenotype of control (*n* = 14) and dexamethasone (*n* = 13) groups, who were airlifted from Delhi (~225 m elevation) to Leh, Ladakh (~3500 m), India, for 3 days. Dexamethasone 4 mg twice daily significantly attenuated the rise in blood pressure, heart rate, pulmonary pressure, and drop in SaO_2_ resulting from high‐altitude exposure compared to control‐treated subjects. Of note, the effect of dexamethasone was substantially greater in females than males, in whom the drug had relatively little effect. Thus, for the first time, this study shows a sex‐biased regulation by dexamethasone of physiologic parameters resulting from the hypoxic environment of high altitude, which impacts the development of high‐altitude pulmonary hypertension and acute mountain sickness. Future studies of cellular contributions towards sex‐specific regulation may provide further insights and preventive measures in managing sex‐specific, high‐altitude‐related disorders.

## ROLE OF THE IMMUNE RESPONSE IN HYPOXIC PULMONARY HYPERTENSION

Rahul Kumar,^1,2^ Neha Chanana,^3^ Biruk Kassa,^1,2^ Kavita Sharma,^3^ Tsering Palmo,^3^ Qadar Pasha,^3,4^ Brian B. Graham^1,2^

^1^Department of Medicine, University of California San Francisco, San Francisco, California, USA; ^2^Division of Pulmonary and Critical Care Medicine, Zuckerberg San Francisco General Hospital, San Francisco, California, USA; ^3^Genomics and Molecular Medicine, CSIR‐Institute of Genomics and Integrative Biology, Delhi, India; ^4^Institute of Hypoxia Research, New Delhi, India

Inflammation is a significant contributor to hypoxic‐pulmonary hypertension (PH) pathogenesis both in humans and experimental animals. We have previously found thrombospondin‐1 (TSP‐1) produced from bone marrow (BM)‐derived interstitial macrophages (IMs) activates tumor growth factor‐β (TGF‐β) to drive hypoxic‐PH. However, understanding the precursor cells that give rise to TSP‐1 + IMs, the molecular mechanisms that cause these cells to travel from the BM to the lungs in hypoxia is not well studied. Dexamethasone is a clinically used drug that blunts acute hypoxic lung diseases in high‐altitude travelers; however, the mechanism by which it protects against hypoxic‐PH is largely unknown. Time‐course‐based flow cytometry analysis using a murine model of 10% FiO_2_ hypoxia exposure revealed a higher number of activated classical (Ly6c^hi^ CCR2^+^) monocytes in BM and intravascular compartments with increasing right ventricular systolic pressure in hypoxia‐exposed wild‐type mice. Quantitative PCR showed higher expression of *Ccr2* in the BM, while enzyme‐linked immunosorbent assay revealed higher levels of the CCR2 ligand, CCL2, in the intravascular compartment and the lungs of the hypoxia‐exposed wild‐type mice. Flow cytometry using *Ccl2* ^*rfp*^ reporter mice and single‐cell analysis on sorted IMs suggested that recruited IMs are a major cellular source of CCL2. Blockade of this pathway using an anti‐CCL2‐neutralizing antibody or transplanting *Ccr2* ^*−/−*^ BM cells into lethally irradiated wild‐type mice protected from hypoxic PH by blocking the recruitment of TSP‐1 + IMs. Dexamethasone treatment phenocopied these effects. These preclinical data were supported by clinical data revealing lower RVSP, TSP‐1, and TGF‐β1 levels in dexamethasone‐treated individuals who traveled from low to high altitudes. Recruitment of classical monocytes derived pathologic TSP‐1 + IMs by modulating the CCR2–CCL2 axis protects from hypoxic‐PH, and may be the mechanism by which dexamethasone protects from acute hypoxic lung disease.

**Funding:** This study was supported by AHA, ATS‐PHA, CMREF, and NIH.

## GENOMICS APPROACHES TO MEGAKARYOCYTE AND PLATELET DISORDERS

Jorge Di Paola

Pediatrics and Molecular Genetics and Genomics, School of Medicine, Washington University, St. Louis, Missouri, USA

Megakaryopoiesis and thrombopoiesis are tightly regulated components of hematopoiesis that result in the production and release of up to 10^11^ platelets daily to maintain a normal concentration of 150–400 × 10^9^ circulating platelets. These cells are required for adequate hemostasis through the formation of a stable clot at sites of blood vessel injury. Thrombocytopenia, traditionally defined as a platelet count of <150 × 10^9^/L, has many inherited and acquired causes, including immune destruction, medication‐induced, aplastic anemia, or as a manifestation of an inherited bone marrow failure syndrome. Interestingly, over the last decade, it has become apparent that a significant proportion of circulating platelets is derived from lung megakaryocytes, although it is uncertain if these megakaryocytes are different from bone marrow‐residing ones. Currently, there are more than 45 genes that have been identified as causative of inherited platelet disorders of number and function. Our laboratory uses genomic approaches to identify the genetic determinants of platelet production and function. Over the last decade, we have been successful in identifying genetic causes of hereditary thrombocytopenias including the discovery of mutations in *NBEAL2* that causes Gray Platelet Syndrome, and the elucidation of a thrombocytopenia with cancer predisposition syndrome caused by mutations in *ETV6*. In collaboration with the Prchal laboratory, we have recently focused our efforts on a highly prevalent autosomal dominant thrombocytopenia in the Muslim population in the Kashmir Valley in the northern Indian subcontinent. A total of 830 voluntary, healthy, male blood donors from the Kashmir Valley were included in the study; they were between 15 and 55 years (median 31 years). Of the donors, 15% had thrombocytopenia (mean platelet count 109.6 compared to 189.9 in controls; *p* ≤ 0.0001) with a statistically significant higher mean platelet volume (12.53 + 0.78 vs. 9.52 + 1.03 fl). The red cell distribution width in thrombocytopenic subjects was higher than in those with normal counts (15.6 + 1.61 vs. 13.22 + 1.36, *p* ≤ 0.001). Hematocrit and other red cell indices were not different in the two groups. Peripheral blood platelet morphology revealed large platelets in all subjects. In a pilot study of seven families, this Kashmiri thrombocytopenia was compatible with autosomal dominant inheritance affecting both males and females. The congenital nature of Kashmiri thrombocytopenia was demonstrated by analyses of 34 consecutive neonates born in Sher‐i‐Kashmir Institute Hospital; among 20 girls and 19 boys, we found 18% (2 male and 5 female) to have low platelet count; the mean platelet count of the affected group, when compared to the unaffected group, were 102.6 versus 234 (*p* ≤ 0.001) respectively. Initial genetic analysis showed no mutations in known genes that cause inherited thrombocytopenia. Recruitment of cases and controls, as well as three‐generation families, is ongoing to identify the genetic cause of the Kashmiri thrombocytopenia.

## THE GENETICS AND EPIGENETICS ARCHITECTURE OF MALADAPTATION TO HIGH ALTITUDE

Aastha Mishra and Qadar Pasha

Cardio Respiratory Disease Unit, CSIR‐Institute of Genomics and Integrative Biology, Mall Road, Delhi, India; Genomics and Molecular Medicine, CSIR‐Institute of Genomics and Integrative Biology, Delhi, India; Institute of Hypoxia Research, New Delhi, India

The effect of high altitude (HA, altitude > 2500 m) on humans is a considerable stress that affects multiple tissues and organs, including the heart, lungs, and brain. As a result, the body starts to tackle hypoxic condition that affects multiple pathways. Some sojourners visiting show better tolerance to hypoxia, but in some, a maladaptive response leading to various forms of acute and chronic HA illnesses are triggered. HA pulmonary edema (HAPE) is one of the acute but severe HA illnesses that develop upon rapid ascent to altitudes above 2500 m in otherwise healthy individuals. Genetic and epigenetic screening of HAPE patients by our group has identified several significantly associated genetic and epigenetic factors that are involved in its pathophysiology. Genes from the pathways, particularly associated with hypoxia response signaling, such as the hypoxia‐inducible factor pathway and vasomodulators, such as the renin–angiotensin–aldosterone system, have attributed the most. In addition to genetics, epigenetic influences were also observed in a few genes. For example, *apelin* rs3761581G and rs2235312T, individually and in combination, and a greater methylation of a CpG island in the 5′‐untranslated region, associated with low levels of apelin‐13 and nitrite in HAPE. A significantly lower methylation distribution was observed in prolyl hydroxylase‐2 (*EGLN1)* in HAPE‐p, which was correlated with upregulated plasma EGLN1 levels (*R* = –0.36, *p* = 0.002) and decreased blood arterial oxygen saturation levels (*R* = 0.34, *p* = 0.004). Along with the differential distribution of the *EGLN1* variant alleles, we could also identify the transcription factors associated with the variants in the healthy and susceptible subjects.

## TELOMERIC TANKYRASE: PHARMACOLOGICAL TARGET FOR HIGH‐ALTITUDE HYPOXIA PATHOPHYSIOLOGY

Arpana Vibhuti,^1^ Manjula Miglani,^1,2^ Qadar Pasha^2^

^1^SRM University, Delhi‐NCR Sonepat, Haryana, India; ^2^CSIR‐Institute of Genomics and Integrative Biology, Delhi, India

Extreme environments at high altitudes (HAs) and cellular exposure lead to the expression of multiple proteins that participate in pathophysiological manifestations. Hypobaric hypoxia at HA generates reactive oxygen species (ROS) that can damage telomeres and disturb normal physiological processes. Telomere complex comprises multiple proteins, of which, tankyrase (TNKS) is actively involved in DNA damage repairs. Understanding the roles of telomeres, telomerase, and TNKS could ameliorate physiological issues experienced at HA. We hence investigated the association of TNKS and telomeres with high‐altitude pulmonary edema (HAPE) to delineate their potential role in HA. Plasma TNKS level was estimated using enzyme‐linked immunosorbent assay, expression of TNKS and relative telomere length were assessed by reverse transcription‐quantitative polymerase chain reaction (RT‐qPCR), and telomerase activity was assessed by the telomere repeat amplification protocol (TRAP) assay. TNKS expression was upregulated by 9.27‐folds in HAPE‐p (*p* = 1.01E − 06) and downregulated in HLs by 3.3‐folds (*p* = 0.02). The telomere length was shorter in HAPE‐p compared to HAPE‐r (*p* = 0.03) and HLs (*p* = 4.25E − 4). The telomerase activity was significantly higher in HAPE‐p compared to both HAPE‐r (*p* = 0.01) and HLs (*p* = 0.001). HAPE‐p had the lowest TNKS levels (0.186 ± 0.031 ng/μl) and the highest telomerase activity (0.0268 amoles/μl). The findings indicate the association of TNKS and telomeres with hypoxia‐induced sicknesses/acclimatization. The plausible findings on TNKS lead to further studies on pharmacological targets for HA hypoxia therapeutics. Thus, a new research avenue on TNKS linked to HA sickness might lead to the discovery of drugs for hypobaric hypoxia.

## HETEROZYGOUS ADVANTAGE OF ANGIOTENSIN‐CONVERTING ENZYME GENE POLYMORPHISM AMONG THE TAWANG MONPA OF EASTERN HIMALAYAN MOUNTAINS OF INDIA

Sudipta Ghosh,^1^ Abigail Bigham^2^ and Tom D. Brutsaert^3^

^1^Department of Anthropology, North‐Eastern Hill University, Shillong, Meghalaya, India; ^2^Department of Anthropology, University of California Los Angeles, California, USA; ^3^Department of Exercise Science, Syracuse University, New York, New York, USA

Angiotensin‐converting enzyme (ACE) plays an important role in the production of angiotensin II and the degradation of bradykinin, two peptides involved in cardiovascular homeostasis. A polymorphism in intron 16 of the human *ACE* gene has been identified in which the presence (insertion, I allele) rather than the absence (deletion, D allele) of a 287 bp fragment is associated with lower serum and tissue ACE activity. Previous studies suggest that this insertion/deletion (I/D) polymorphism has a significant genetic influence on high‐altitude native populations in their adaptation to hypobaric hypoxia. An excess of the I‐allele has been reported in high‐altitude natives from Peru and Ladakh. We characterized the *ACE* genotype distribution among the Tawang Monpa with special emphasis on its association with arterial oxygen saturation (SaO_2_), arterial blood pressure, haemoglobin (Hb) concentration, and ventilatory measures. Unlike high‐altitude natives from Peru and Ladakh, who exhibit high frequencies of II homozygotes, Tawang Monpa shows a significantly high frequency of ID heterozygotes (*p* < 0.0001). In addition, we did not identify a significant association between *ACE* gene polymorphism and arterial blood pressure, oxygen saturation at rest, vital capacity, or [Hb] concentration in this population. In conclusion, our results suggest that *ACE* I/D gene polymorphism may have a heterozygous advantage for certain native populations for their adaptation to high‐altitude hypoxia. Moreover, the association between *ACE* gene and resting SaO_2_ is not universal across native populations living under hypobaric hypoxia.

## CHALLENGES IN THE TREATMENT OF HEAD AND NECK CANCER IN INDIA: NOVEL THERAPEUTIC APPROACHES TO TACKLE HNSCC

Palak Parashar, Vibha Tandon

Centre for Molecular Medicine, Jawaharlal Nehru University, New Delhi, India

Head and neck squamous cell carcinoma treated with radiation suffers from xerostomia caused by damage to salivary glands. In this study, we used patient‐derived xenograft mice model to investigate the effect of a novel bisbenzimidazole previously synthesized in our laboratory as radiomodulator and salivary gland cytoprotectant to evaluate real possibilities of reducing the incidence and severity of xerostomia in head and neck squamous cell carcinoma patients. The rate of wound closure decreased to 3.62, 3.83, and 4.1 µm for dimethylamine (DMA) + 4 Gy radiation in comparison to 7.64, 7.73, and 8.69 µm for 4 Gy radiation alone at 24, 48, and 72 h. We observed a significant increase in saliva secretion in HK‐1 xenograft treated with 50 mg/kg DMA and 50 mg/kg DMA+4 Gy radiation in patient‐derived xenograft treated with 25 mg/kg DMA and 25 mg/kg DMA + 4 Gy radiation. A significantly improved mean survival rate of 83% and 100% was observed in xenografts treated with 50 mg/kg DMA + 4 Gy radiation and 50 mg/kg DMA for 5 consecutive days compared to 33% in those treated with 200 mg/kg amifostine + 4 Gy radiation for 5 days. After DMA treatment, the number of Ki‐67 proliferating stained cells significantly increased in the spleen, intestines, and lungs compared to the tumor tissue, suggesting that DMA acts as a radioprotector for normal tissues. We also investigated the relationship between integrin‐β3 and therapeutic outcomes through single‐photon emission computed tomography. The levels of aspartate aminotransferase and alkaline phosphatase decreased after DMA treatment, indicating that DMA along with radiation prevents hepatotoxicity and the likelihood of tumor metastasis.

## CANCER AND PULMONARY HYPERTENSION: LEARNING LESSONS AND REAL‐LIFE INTERPLAY

Rajkumar Savai

Max Planck Institute for Heart and Lung Research, Member of the German Center for Lung Research (DZL), Member of the Cardio‐Pulmonary Institute (CPI), Bad Nauheim, Germany; Department of Internal Medicine, Member of the DZL, Member of CPI, Justus Liebig University, Giessen, Germany; Institute for Lung Health, Justus Liebig University, Giessen, Germany

Lung cancer and its comorbidities such as chronic pulmonary obstructive pulmonary (COPD) or pulmonary fibrosis (PF) share similar risk factors. In this context, cigarette smoking is the most notable one to mention. Smoking causes inflammation and changes the lung organ microenvironment. Additionally, lung cancer also shares similar molecular signal pathways with diseases like COPD, PF, or pulmonary hypertension (PH). While many studies have been conducted on these diseases alone, the complex interplay of lung cancer and its comorbidities is only now being better understood. There are first reports, for instance, showing that PH as a comorbidity strongly limits life expectancy in lung cancer patients. Tumor progression and lethality are crucially influenced by tumor cells and their surrounding tumor microenvironment (TME). Improving our understanding of the influence of common lung cancer comorbidities on the TME will be a key to developing more enhanced lung cancer therapies in the near future. This presentation compares lung cancer and pulmonary hypertension in view of the hallmarks of cancer with an emphasis on immune/inflammatory aspects of the disease to find novel therapies.

## CROSSTALK BETWEEN HYPOXIA AND ESTROGEN SIGNALING IN THE PULMONARY VASCULATURE AND RIGHT VENTRICLE

Tim Lahm

National Jewish Health and University of Colorado, Denver, Colorado, USA

17β‐Estradiol (E2) attenuates hypoxia‐induced pulmonary hypertension (HPH) through estrogen receptor (ER)‐dependent effects, including inhibition of hypoxia‐induced pulmonary artery endothelial cell (PAEC) proliferation; however, the underlying mechanisms remain unknown. We hypothesized that the protective effects of E2 in HPH are mediated through hypoxia‐inducible factor 1α (HIF‐1α)‐dependent increases in ERβ expression. We also hypothesized that estrogenic pathways are a component of lung and right ventricle (RV) transcriptome responses in an adaptative HPH model. Sprague–Dawley rats and ERα‐ or ERβ‐knockout mice were exposed to hypobaric hypoxia for 2–3 weeks. Effects of hypoxia were also studied in primary rat or human PAECs. We performed transcriptome analyses in a novel rat model of perinatal hypoxia exposure and adaptative HPH. Hypoxia increased the expression of ERβ, but not ERα, in the lungs of HPH rats as well as in rat and human PAECs. ERβ messenger RNA time‐dependently increased in PAECs exposed to hypoxia. Normoxic HIF‐1α/HIF‐2α stabilization increased PAEC ERβ, whereas HIF‐1α knockdown decreased ERβ abundance in hypoxic PAECs. In turn, ERβ knockdown in hypoxic PAECs increased HIF‐2α expression, suggesting a hypoxia‐sensitive feedback mechanism. ERβ knockdown in hypoxic PAECs decreased the expression of the HIF inhibitor prolyl hydroxylase 2 (PHD2), whereas ERβ activation increased PHD2 and decreased both HIF‐1α and HIF‐2α, suggesting that ERβ regulates the PHD2/HIF‐1α/HIF‐2α axis during hypoxia. Whereas hypoxic wild‐type or ERα‐knockout mice treated with E2 demonstrated less pulmonary vascular remodeling and decreased HIF‐1α after hypoxia compared with untreated hypoxic mice, ERβ‐knockout mice exhibited increased HIF‐2α and an attenuated response to E2 during hypoxia. In transcriptome analyses in rats with perinatal hypoxia exposure with adaptive lung and RV remodeling, we identified estrogenic signaling as a prominently upregulated pathway. Thus, we identified a novel mechanism whereby hypoxia, via HIF‐1α, increases ERβ expression and the E2–ERβ axis targets PHD2, HIF‐1α, and HIF‐2α to attenuate HPH development. Estrogenic signaling is a component of transcriptomic alterations that may mediate adaptation to chronic hypoxia.

## THE ROLE OF WNT SIGNALING IN THE PATHOGENESIS OF PULMONARY VASCULAR DISEASE

Vinicio A. de Jesus Perez

Division of Pulmonary, Allergy and Critical Care Medicine, Stanford University Medical Center, Stanford, California, USA

Pulmonary arterial hypertension (PAH) is characterized by the loss of microvessels. The Wnt pathways control pulmonary angiogenesis, but their role in PAH is incompletely understood. We hypothesized that Wnt activation in pulmonary microvascular endothelial cells (PMVECs) is required for pulmonary angiogenesis, and its loss contributes to PAH. Lung tissue and PMVECs from healthy and PAH patients were screened for Wnt production. Healthy PMVECs demonstrated >6‐fold Wnt7a expression during sprouting angiogenesis that was absent in PAH PMVECs and vascular lesions. Wnt7a expression correlated with the formation of tip cells, a highly migratory endothelial phenotype critical for angiogenesis. We found that PAH PMVECs demonstrated reduced VEGF‐induced tip cell formation as evidenced by reduced filopodia formation and motility, which could be partially rescued by recombinant Wnt7a. We discovered that Wnt7a promotes VEGF signaling by facilitating phosphorylation of Y1175 tyrosine residues in VEGFR2 through ROR2, a Wnt‐specific receptor. We found that ROR2 knockdown mimics Wnt7a insufficiency and prevents recovery of tip cell formation with Wnt7a stimulation. While there was no difference between wild‐type and endothelial‐specific Wnt7a−/− mice under either chronic hypoxia or Sugen hypoxia (SuHx), global Wnt7a+/− mice in chronic hypoxia demonstrated higher pulmonary pressures and more severe right ventricular and lung vascular remodeling. Surprisingly, Wnt7a−/− animals died soon after induction and exhibited severe cardiopulmonary defects. We have identified Wnt7a as a novel angiogenic factor that plays a pivotal role in pulmonary angiogenesis by promoting VEGF‐A‐mediated tip cell formation. Targeting Wnt7a could promote the recovery of small vessels in PAH.

## STUDY ON THE INVOLVEMENT OF POLY (ADP) RIBOSE POLYMERASE‐1 IN LUNG ENDOTHELIAL FUNCTIONS IN PULMONARY HYPERTENSION

Mohammad Shafiq and Kashif Hanif

Division of Pharmacology, CSIR‐Central Drug Research Institute, Lucknow, India

In pulmonary hypertension (PH), increased inflammation and oxidative/nitrosative stress cause DNA damage, activating poly (ADP‐ribose) polymerase‐1 (PARP‐1). It has been reported that PARP‐1 inhibition is protective in PH and is associated with right ventricle hypertrophy. However, the role of PARP‐1 in pulmonary artery endothelial dysfunction warrants exploration. Therefore, the current study investigated the role of PARP‐1 in endothelial dysfunction associated with PH. Human pulmonary artery endothelial cells (HPAECs) were exposed to hypoxia (1% O_2_), and PARP‐1 inhibition was achieved via small interfering RNA (siRNA) (60 nM). For in vivo studies, male Sprague–Dawley rats were administered monocrotaline (MCT) (60 mg/kg, subcutaneously, once) to induce PH in 35 days, and PARP‐1 inhibitor, 1,5‐isoquniolinediol (ISO) (3 mg/kg, intraperitoneally), was administered daily. Western blot analysis, qualitative reverse transcription‐PCR, immunohistochemistry, flow cytometry, Comet assay, and enzyme‐linked immunoassays were performed to study the involvement of PARP‐1 in pulmonary endothelial dysfunction in PH. Inhibition of PARP‐1 reduced the increased expression and activity of PARP‐1 in the hypoxic HPAECs and MCT‐treated rats. PARP‐1 inhibition by siRNA decreased inflammation, VEGF expression, and tubule formation, but improved mitochondrial function in hypoxic HPAECs. PARP‐1 inhibition increased apoptosis and reduced proliferation and DNA repair as shown by the Comet assay in hypoxic HPAEC. In in vivo studies, PARP‐1 inhibitor ISO significantly increased the pulmonary vascular reactivity and expression of phospho‐endothelial nitric oxide synthase in MCT‐treated rats. Results showed that PARP‐1 plays a crucial role in PH and its inhibition improves pulmonary endothelial functions in PH.

## MONOCYTES WITH TIBETAN‐SPECIFIC PHD2^D4E;C127S^ MUTATION DISPLAY PROTECTION AGAINST VIRAL INFECTION UNDER A HYPOXIC ENVIRONMENT, BUT ARE SUSCEPTIBLE TO INFECTION UNDER NORMOXIA

Riya Ghosh, Nishith M. Shrimali, Prasenjit Guchhait

Regional Centre for Biotechnology, National Capital Region Biotech Science Cluster, Faridabad, India

Tibetans carry adaptive mutations in *EGLN1* (encodes prolyl hydroxylase 2 [PHD2], a negative regulator of hypoxia‐inducible factor [HIFα]) and *EPAS1* (encodes HIF2α) that protect them from polycythaemia in the hypoxic environment of high altitude. Recently, we have described that Tibetans carrying homozygous PHD2^D4E;C127S^ mutation are protected from hypoxia‐induced inflammatory complications. Leukocytes including monocytes with homozygous PHD2^D4E;C127S^ mutation displayed less secretion of cytokines such as IL‐6 and IL‐1β as well as decreased chemotactic response, corresponding to the downmodulated expression of related signaling molecules such as P65, JUN, STAT1, ATF2, and CXCR4. In the present study, we describe that the PHD2^D4E;C127S^ monocytes are protected from viral infections such as dengue and Japanese encephalitis under a hypoxic environment. In contrast, these cells are susceptible to infection in normoxia. It is known that viruses utilize Akt signaling for their replication and propagation in host cells. We describe that Akt phosphorylation plays a crucial role in the above mechanism.

## ROLE OF LOCAL COMPLEMENT SIGNALING IN HYPOXIA‐INDUCED LUNG INTERSTITIAL MACROPHAGE REPROGRAMMING

Sushil Kumar,^1^ Ram Raj Prasad,^1^ S. Chaudhary,^2^ Min Li,^1^ Hui Zhang,^1^ Suzette Riddle,^1^ Maria G. Frid,^1^ B. Alexandre McKeon,^1^ Subhash C. Yadav,^2^ Hu Cheng‐Jun,^1^ Kirk C. Hansen,^3^ Kurt R. Stenmark^1^

^1^Cardiovascular Pulmonary Research Laboratories, Department of Pediatrics and Medicine, University of Colorado, Anschutz Medical Campus, Aurora, Colorado, USA; ^2^Department of Biochemistry and Molecular Genetics, University of Colorado, Anschutz Medical Campus, Aurora, Colorado, USA; ^3^Department of Anatomy, All India Institute of Medical Sciences, New Delhi, India.

Vascular inflammation is currently recognized as an important prognostic determinant in all forms of pulmonary hypertension (PH). Elevated numbers of perivascular monocytes/macrophages with distinct activation profiles have been reported in experimental animal models of hypoxia and in humans with PH. Locally produced complement components by distal pulmonary artery cells can either act directly and/or through small extracellular vesicles (sEVs) and have function‐modulating effects on target cells, including macrophages. We have previously shown a significant role for systemically derived complement activation in the macrophage accumulation and vascular remodeling observed in hypoxic PH. New data support the idea that local cell‐derived complements can contribute to an inflammatory response in various tissues in the body. However, the role of locally produced complements in facilitating vascular inflammation in PH remains undefined. Pulmonary adventitial fibroblasts were isolated from calves with severe PH (PH‐Fibs) and age‐matched controls (CO‐Fibs). We determined the transcriptomics profile of CO‐Fibs and PH‐Fibs by performing RNAseq. We observed significant upregulation in several complement‐associated gene including C3, CFB, C4, CFD, and C7, as well as proinflammatory associated genes, including IL‐6 and IL1B in PH‐Fibs compared to CO‐Fibs. Protein analysis of CO‐Fibs and PH‐Fibs by immunoblot revealed full length (inactive form) as well as various activated fragments of C3 protein (C3c and C3d) with the activated fragments of C3 markedly being increased in PH‐Fibs compared to CO‐Fibs, suggesting local production and activation of C3 protein in fibroblast. We isolated sEVs from CO‐Fibs and PH‐Fibs conditioned media and found sEVs from PH‐fibs stimulated inflammatory and metabolic reprogramming in bone marrow‐derived macrophages (BMDM). Proteomic analysis of sEVs revealed a marked increase of C3 protein in PH‐Fib‐sEVs compared to CO‐Fib‐sEVs. The gene set enrichment analysis of the transcriptome of the PH‐fibs as well as proteome of sEVs derived from PH‐Fibs compared with CO‐Fibs revealed enrichment of complement and coagulation cascades with the highest score. We found that sEVs from C3‐depleted PH‐Fibs failed to stimulate BMDM inflammatory gene expression and metabolic reprogramming. Overall, our data suggested C3 protein of fibroblast can be released directly in their microenvironment and/or through packaging in sEVs. The local production of complement and complement‐containing sEVs, derived from fibroblast of calves with hypoxia‐induced PH, induce expression of proinflammatory mediators and metabolic reprogramming of macrophages.

**Funding:** This study was supported by grants from the NIH P01HL152961 and P01HL014985 to 593 (to K. R. S.), and DOD W81XWH2010249 and W81XWH1910259 (to K. R. S.).

## TRANSESOPHAGEAL ECHOCARDIOGRAPHIC ASSESSMENT OF HEMODYNAMIC CHANGES DURING LAPAROSCOPIC SURGERIES AT HIGH ALTITUDE

Tsering Morup,^1^ Krishna P. Gourav,^2^ Goverdhan Dutt Puri,^2^ Sunder L. Negi^2^

^1^Sonam N. Memorial. Hospital, Leh, Ladakh, India; ^2^Postgraduate Institute of Medical Education and Research, Chandigarh, India

Anaesthesia at high altitude is challenging due to vast variations in physiology, which are further complicated by the positioning and pneumoperitoneum during laparoscopic surgeries. These changes can be better understood and managed with the help of echocardiography. Here, we demonstrate the effect of laparoscopy on hemodynamics with the help of transesophageal echocardiography (TEE) at high altitudes in three patients. Three ASAI patients (Patient 1, laparoscopic cholecystectomy; Patient 2, laparoscopic vaginal hysterectomy; Patient 3, laparoscopic hernioplasty with mesh repair) who underwent laparoscopic surgeries at an altitude of 3500 m were studied. Various parameters were measured by TEE, which included left ventricular ejection fraction (EF), left ventricular outflow tract (LVOT), velocity–time integral (VTI), cardiac output (CO), E/A, E/e′, tricuspid annular plane excursion (TAPSE), and pulmonary artery acceleration time. The mean arterial pressure (MAP), heart rate, oxygen saturation, and end‐tidal carbon dioxide were also measured. These parameters were recorded at 10 time points: before induction of anaesthesia (T1), before insufflation (T2), after insufflation (T3), 5 mmHg pneumoperitoneum (T5), 14 mmHg pneumoperitoneum (T6), 10 min after 14 mmHg pneumoperitoneum (T7), 20 min after 14 mmHg pneumoperitoneum (T8), 30 min after 14 mmHg pneumoperitoneum (T9), and 5 min after desufflation (T10). We observed a decrease in MAP, LVOT VTI, and CO after pneumoperitoneum when associated with the reverse RT position and an increase in MAP, LVOT VTI, and CO when associated with the Trendelenburg position. The right ventricular systolic function measured by TAPSE, left ventricular EF, and LV diastolic function remained the same throughout the procedure in all three patients. Pulmonary artery acceleration time gradually decreased after pneumoperitoneum in all three patients but stayed in the normal range throughout the procedure. The results of our study are consistent with previous studies performed at sea level. The observations showed that laparoscopic surgeries may be safely performed in healthy individuals at high altitudes. However, the study was limited by a small sample size and was done only on healthy subjects.

## ROLE OF MAGNETIC RESONANCE IMAGING IN UNDERSTANDING THE PATHOBIOLOGY OF PULMONARY ARTERIAL HYPERTENSION

Mahesh Kappanayil

Department of Pediatric Cardiology, Amrita Institute of Medical Sciences, Kochi, Kerala, India

Pulmonary hypertension, irrespective of the underlying etiopathogenesis, is a result of anatomical and physiological perturbations in the lung vasculature and function. Invasive right heart catheterization is the gold standard for diagnosing and objectively measuring pulmonary artery hypertension (PAH). However, there is tremendous value to careful clinical assessment and noninvasive cardiovascular imaging in the assessment and deeper understanding of PAH. The multimodality approach to cardiovascular imaging proves useful in answering key questions regarding etiology of PAH, pathophysiological changes resulting from PAH, cardiovascular remodeling, and the anatomical and physiological responses to drug therapy. Echocardiography has long been in widespread use as a relatively simple, inexpensive, noninvasive tool for objectively measuring pulmonary artery pressures and predicting pulmonary vascular resistances. Computerized tomography scans can give excellent information about the anatomy of the pulmonary vascular bed. Cardiac magnetic resonance imaging (MRI) is a novel modality and is revolutionizing cardiac imaging. It is finding an increased role in the assessment of PAH. Using a powerful set of diverse imaging tools based on the principle of magnetic resonance, cardiac MRI provides unprecedented 3D visualization of cardiopulmonary structures, while providing a unique, deep insight into flows and functions, thereby adding tremendously to the understanding of the pathophysiology of PAH and in measuring response to treatment. An intelligent integration of these noninvasive modalities can provide vital insight into understanding and managing the pulmonary vascular disease. This talk will focus on the science behind the imaging techniques and how MRI can be effectively leveraged for research as well as clinical applications in pulmonary hypertension and associated physiologies.

## HIGH ALTITUDE AND MALADIES: EXPERIENCES AND OBSERVATIONS OF A LOCAL SURGEON

Padma Deskit

Sonam N. Memorial Hospital, Leh, Ladakh, India

Visit to a high‐altitude place with its hypoxic, hypobaric, cold, and windy hostile environment poses various physiological and biochemical challenges to the human body. While we normally correlated high‐altitude maladies with more popular and prevalent high‐altitude pulmonary edema (HAPE) and high‐altitude cerebral edema (HACE), unfortunately, high altitude causes multisystemic derangement well beyond the ambit of any particular speciality. While HAPE and HACE are acute, and more prevalent, vascular and other complications due to high altitude are insidious and less talked about, but equally life‐threatening. In the preceding 5–6 years, we witnessed and managed an array of ailments in our surgical field, which could be attributed to high altitude. With this presentation, we would like to draw the attention of scholars, researchers, and clinicians to the high‐altitude‐related surgical maladies. Ours is a retrospective study of all patients with surgical ailments due to high altitude who were managed at Sonam Norboo Memorial Hospital and Army Hospital, Leh from 2017 to 2022. Surgeries were conducted on patients with different conditions: (i) *vascular complications*: high‐altitude‐related vascular complications were analysed. A total of 10 patients were managed, which included four cases of mesenteric ischemia, one case of portal venous thrombosis, one case of testicular infarct, and four cases of splenic infarct, all of them presented as a surgical emergency; (ii) *duodenal ulcer perforation*: surgeries were performed on a total of 50 patients of duodenal ulcer perforation in the past 5 years. Of these patients, 40 were nonlocals who show that high altitude predisposes individuals, leading to duodenal ulceration and gastrointestinal perforation; (iii) *skin cancers*: a total of 35 patients with skin cancers primarily those residing at high altitudes were included in the study. Surgical procedures were followed to manage the patients. Observations indicated that highlanders remain at increased risk of developing skin cancers. With the advancement of technologies in communication, particularly in high‐altitude areas like Ladakh, India, which is considered to be an important holiday destination, the world needs to be made more aware of all possible health‐related maladies at high altitudes. Such knowledge may help in developing suitable strategies to ensure better health care for sojourners as well as highlanders.

## GROWTH AND DEVELOPMENT TRENDS OF LADAKHI NEWBORN INFANTS AT HIGH ALTITUDE: A LONGITUDINAL STUDY OF NEWBORN THROUGH EARLY CHILDHOOD IN LADAKH, NORTHERN INDIA (2015–2018)

Tsering Norboo

Ladakh Institute of Prevention, Leh, Ladakh, India

Children born at high altitudes are known to have low birth weight and slow growth. This study aims to find out if the same holds good for children born in Ladakh at an altitude of 2700–4900 m. By following the growth of 3825 infants born at a single center, the SNM Hospital Leh (alt. 3500 m) between April 2015 and April 2018, for 2 years, we made an attempt to determine, if the growth and development at high altitudes are affected by hypoxic and adaptive process or other socioeconomic factors like nutrition, cultural feeding practices, or infections are involved. A user‐friendly “mother and child handbook” as recommended by World Health Organization in 1998 was used for documentation as a single tool for mother and child health. Anthropometric measurements soon after birth, every 3 months during the first year, half‐yearly in the second year, and once every year thereafter till 5 years was done. The study indicates that despite near‐normal length for age at birth, a significant difference between Ladakhi infants and WHO standards emerged by the 1‐year mark. On average, Ladakhi males recovered from low birth weight by the 1‐year mark, while the females (near‐normal weight on birth) fell below WHO by the 1‐year mark. Differences in length and weight between full‐term and preterm infants remained significant until 13 months. In conclusion, it may be inferred that the near‐normal length and weight of Ladakhi infants at birth is suggestive of high‐altitude ancestry partial adaptation, possibly by an increase in uterine artery diameter in mid‐pregnancy facilitating improved oxygen delivery to the placenta and fetus. Notable is the decline in length and weight soon after birth with a catchup period of 9–13 months. This may be possibly due to lack of feeding practices, nutrition of mother and child, and frequent infections.

## ROLE OF PHOSPHORYLATION IN SURVIVAL AND VIRULENCE OF PATHOGENIC BACTERIA

Yogendra Singh

Department of Zoology, University of Delhi, Delhi, India

In living cells, hundreds of protein modifications have been identified, such as phosphorylation, methylation, acetylation, ADP‐ribosylation, glycosylation, and so on, which results in the diversification of protein functions. Among these modifications, protein phosphorylation is known to be the most common modification that regulates multiple processes, such as cell division, metabolic pathways, stress response, sporulation, and even virulence in pathogens. These events are regulated by specific proteins, such as kinases and phosphatases, which are often essential for the survival and virulence of pathogenic bacteria. We showed that a secretory tyrosine phosphatase in *Mycobacterium tuberculosis* (Mtb) is essential for full virulence and the mutant strain lacking this enzyme was about 100‐fold less virulent. In addition, we also demonstrated the critical role of a lone serine/threonine phosphatase in the survival of Mtb. While Ser/Thr phosphatase is essential for survival, tyrosine phosphatase is required for virulence of Mtb. In another pathogenic bacterium, *Bacillus anthracis*, we reported the role of protein Ser/Thr kinases and phosphatases in the regulation of bacterial chain length and toxin synthesis that are required for virulence of the pathogen. A mutant strain of a Ser/Thr kinase, PrkC, resulted in shortening of bacterial chain length, reduced cell wall thickness, and multiseptation. Recently, we identified a novel serine/threonine phosphatase, which we named PrpN in *B. anthracis* and observed that it controls sporulation and toxins production. In brief, phosphorylation is essential for many cellular functions, including the virulence of many pathogens.

## EXERCISE‐INDUCED PULMONARY VASCULAR DISTENSIBILITY IN HEALTHY VERSUS ATHLETIC SUBJECTS

Marine Carpentier,^1^ Yoshiki Motoji,^1^ Kevin Forton,^2^ Nicolas Selvais,^2^ Martin Chaumont,^2^ Vitalie Faoro^1^

^1^Cardiopulmonary Exercise Physiology Laboratory, Faculty of Motorskills Sciences, Université Libre de Bruxelles, Brussels, Belgium; ^2^Department of Cardiology, Erasmus University Hospital, Brussels, Belgium

Exercise is associated with an increase in pulmonary arterial pressure (PAP) with cardiac output (Q) elevation. However, because of the distensibility of the arteriolar pulmonary vasculature, the mean PAP (mPAP)–Q relationship is not strictly linear. A curvilinear model allows the calculation of the distensibility coefficient *α*, as the percentage change of vessel diameter per mmHg of mPAP increases. It remains unknown how much endurance training affects the *α* distensibility coefficient of the pulmonary circulation. We hypothesize that greater pulmonary vascular distensibility *α* allows for a higher pulmonary vascular reserve and allows a higher Qmax and thus a higher aerobic exercise capacity (VO_2_max), probably observable in trained athletes. Thirty‐four male volunteers, nonsmokers, and free of cardiovascular or lung disease participated in our study. Of these subjects, 17 professional football players (24 ± 3 years old, body mass index 24 ± 2, VO_2_max 41.3 ± 5.4 ml/min/kg) were compared to body dimension and age‐matched sedentary volunteers (VO_2_max 34.5 ± 5.5 ml/min/kg). All underwent a semirecumbent cycloergometer incremental cardiopulmonary exercise test (CPET) with stress echocardiography for noninvasive PAP, left and right atrial pressure, and Q measurements. Pulmonary distensibility *α* index was calculated from multipoint mPAP–Q plots from rest to maximal exercise. The results of the present study confirmed the hypothesis as athletes exhibited a significant increase of the *α* distensibility coefficient in athletes versus controls (1.37 ± 0.41 vs. 1.02 ± 0.31%/mmHg, *p* = 0.017). Therefore, endurance athletes have a greater distensibility of the pulmonary arteriolar vessels as compared to sedentary people, which might contribute to developing higher Qmax and enhancing VO_2_max.

## SEX‐DERIVED ATTRIBUTES CONTRIBUTING TO SARS‐COV‐2 MORTALITY

Neha Chanana,^1,2^ Tsering Palmo,^1^ Kavita Sharma,^1^ Rahul Kumar,^3^ Brian B. Graham,^3^ Qadar Pasha^1,4^

^1^Department of Genomics and Molecular Medicine, CSIR‐Institute of Genomics and Integrative Biology, Delhi, India; ^2^Current affiliation: Department of Biochemistry, Jamia Hamdard, New Delhi, India; ^3^Department of Medicine, University of California San Francisco, San Francisco, CA, USA; ^4^Institute of Hypoxia Research, New Delhi, India

Epidemiological data on coronavirus disease 2019 (COVID‐19) mortality indicate that men are more prone to die of SARS‐CoV‐2 infection than women, but biological causes for this sexual dimorphism are unknown. We discuss the prospective behavioral and biological differences between the sexes that could be attributed to this sex‐based differentiation. The female sex hormones and the immune stimulatory genes, including Toll‐like receptors, interleukins, and micro‐RNAs present on X‐chromosome, may impart lesser infectivity and mortality of the SARS‐CoV‐2 in females over males. The sex hormone estrogen interacts with the renin–angiotensin–aldosterone system, one of the most critical pathways in COVID‐19 infectivity, and modulates the vasomotor homeostasis. Testosterone on the contrary enhances the levels of the two most critical molecules, angiotensin‐converting enzyme 2 and the transmembrane protease serine‐type 2, transcriptionally and posttranslationally, thereby increasing viral load and delaying viral clearance in men as compared with women. We propose that modulating sex hormones, either by increasing estrogen or antiandrogen, may be a therapeutic option to reduce mortality from SARS‐CoV‐2.

## TELOMERE–TELOMERASE–TANKYRASE TRIO IN ACTION AT HIGH ALTITUDE: A PERFECT SYMPHONY FOR PROTECTION AGAINST HIGH‐ALTITUDE DISORDERS

Manjula Miglani,^1,2^ Qadar Pasha,^2,3^ Arpana Vibhuti^1^

^1^SRM University, Haryana, India; ^2^CSIR‐Institute of Genomics and Integrative Biology, Delhi, India; ^3^Institute of Hypoxia Research, New Delhi, India

Multiple proteins are expressed that participate in the pathophysiological manifestations upon cellular exposure to extreme environments. Telomeres are prone to damage by the reactive oxygen species and ultraviolet rays present at high altitudes (HAs). Telomere complex constitutes multiple proteins, of which tankyrase (TNKS) and telomerase are actively involved in DNA damage repair. Telomere–telomerase coordination is maintained by TNKS and telomere repeat factors (TRFs), which are positive and negative regulators of telomere length, respectively. Multiple reports on TNKS indicate their possible role in cancer and other hypoxic diseases; however, its role in high‐altitude sicknesses remains elusive. The present study investigated the polymorphism of TNKS gene, expression, and biochemical levels in three well‐defined groups, namely, high‐altitude pulmonary edema patients (HAPE‐p), HAPE‐resistant sojourners (HAPE‐r), and healthy highlanders (HLs) comprising 200 samples, each. Genotyping was performed using PCR‐restriction fragment length polymorphism, expression of *TNKS* and *TRF* were assessed by quantitative reverse transcription‐PCR, and the protein level was estimated using enzyme‐linked immunosorbent assay (ELISA) and correlations were analysed. In TNKS polymorphism, heterozygotes rs7015700GA were prevalent in HLs compared to the HAPE‐p and HAPE‐r. The plasma TNKS was significantly decreased in HAPE‐p than HAPE‐r (*p* = 0.006). The expression of *TNKS* was upregulated 9.27‐folds in HAPE‐p (*p* = 1.01E − 06) and downregulated in HLs by 3.3 folds (*p* = 0.02). The telomere length was shorter in HAPE‐p compared to HAPE‐r (*p* = 0.03*)* and HLs (*p* = 4.25E − 4). The telomerase activity was significantly higher in HAPE‐p compared to both HAPE‐r (*p* = 0.01) and HLs (*p* = 0.001). The findings of the study indicate the association of *TNKS* and telomerase with telomeres towards protection against HA disorders.

## THE MISSENSE VARIANT RS5370 G > T OF ENDOTHELIN‐1 IS ASSOCIATED WITH HIGH‐ALTITUDE PULMONARY EDEMA AND ADAPTATION AT HIGH ALTITUDE

Tsering Palmo,^1^ Neha Chanana,^1,2^ Bilal Ahmed Abbasi,^1^ Kavita Sharma,^1^ Mohammed Faruq,^1^ Tashi Thinlas,^3^ Malik Z. Abdin,^4^ Qadar Pasha^1,5^

^1^CSIR‐Institute of Genomics and Integrative Biology, Mall Road, Delhi, India; ^2^Current address: Department of Biochemistry, Jamia Hamdard, New Delhi, India; ^3^Sonam N. Memorial Hospital, Leh, Ladakh, India; ^4^Department of Biotechnology, Jamia Hamdard, New Delhi, India; ^5^Institute of Hypoxia Research, New Delhi, India

Endothelin 1 (*EDN1*) encodes a potent endogenous vasoconstrictor, ET1, to maintain vascular homeostasis. The *EDN1* polymorphism rs5370 *G*/*T* has been strongly associated with cardiopulmonary diseases. This study investigated the impact of rs5370 polymorphism in high‐altitude pulmonary edema (HAPE) disorder and adaptation physiology. In a well‐characterised case–control study of high‐ and low‐altitude populations comprising 310 samples each of HAPE‐patients (HAPE‐p), HAPE‐free controls (HAPE‐f), and native highlanders (HLs), the rs5370 polymorphism was genotyped, and the gene expression and plasma level of EDN1 were evaluated. The functional relevance of each allele was investigated in the human embryonic kidney 293 cell line after exposure to hypoxia and in presence of hypoxia‐inducible factor‐1α (HIF1α), and also by applying the bioinformatics tools. The *T* allele was more prevalent in HAPE‐p when compared with HAPE‐f and HLs (*p* = 0.05 and <0.0001, respectively). The gene expression was upregulated and the biolevel was significantly elevated in HAPE‐p when compared with controls (*p* < 0.0001). The *T* allele compared to *G* allele was associated with the increased level of ET‐1 in the three study groups and increased endogenous ET‐1 level in cells exposed to hypoxia and treated with HIF1α (*p* < 0.05). The in silico studies supported the deleterious effect of the *T* allele on the structural integrity and function of the protein. The rs5370 *T* allele is associated with the increased concentration of ET‐1 both in vivo and in vitro, establishing it as a potent marker in adaptation and maladaptation to high‐altitude environment.

## MECHANISTIC BASIS OF THE FIDELITY OF HISTONE H2B MONOUBIQUITINATION REACTION BY RAD6 AND BRE1

Pawan Yadav,^1^ Rushna Wazahat,^1^ Manish Gupta,^2^ Pankaj Kumar^2^

^1^Department of Biochemistry, School of Chemical and Life Sciences, Jamia Hamdard University, New Delhi, India; ^2^Department of Medicine, Johns Hopkins University, Baltimore, Maryland, USA

Monoubiquitination at histone H2B‐K123 in yeast (H2BK120 in humans) plays an important role in gene regulation. It is a highly conserved process that involves E2‐conjugating enzyme (Rad6) and E3 ligase (Bre1). Ubiquitination at histone H2B leads to structural changes in nucleosome core particle (NCP), which allows other factors to bind, helping in gene activation, silencing, DNA repair, histone methylation, and so on. Previous studies have highlighted the role of Rad6 and Bre1 in monoubiquitination at the histone H2BK123 site in yeast. However, the mechanistic basis of Rad6/Bre1‐dependent histone H2B monoubiquitination was unknown. In our present study, we aim to characterize the role of a C‐terminal acidic tail of Rad6 and its interaction with Bre1 for site‐specific H2B monoubiquitination. Through solution‐based structural biology, in vitro assays, and binding studies, we have shown that Rad6 acidic tail binds to positively charged surface on H2B restricting the access of Rad6 to 15 A° radii on NCP surface. This allows only promiscuous ubiquitination of various lysine residues present in the vicinity without a Bre1. On the other hand, RBD (Rad6‐binding domain) along with the RING domain in Bre1 inhibits the unspecific transfer of ubiquitin by Rad6 on histones by modulating the dynamics of the acidic tail and restricting only H2BK123 monoubiquitination. Also, we have found that deletion of acidic tail or mutation in the positively charged region of H2B leads to disruption of physical interaction between histones and E2 enzyme. Taken together, we aim to reveal the mechanistic basis of constrains in E2:E3 and E2:NCP recognition that ensures the fidelity of histone H2B monoubiquitination reaction.

## PREVALENCE OF HYPOVITAMINOSIS D AMONG PEOPLE LIVING AT HIGH ALTITUDE IN LEH, LADAKH, INDIA

Syed Mohd, Aastha Mishra, Swati Sharma, Shankar Chanchal, Yasir Khan, Mohammad Zahid Ashraf

Jamia Millia Islamia, New Delhi, India

Hypovitaminosis D is a highly prevalent condition worldwide; however, there is little information about its prevalence in apparently healthy people living at high altitudes (HAs). Therefore, it is important to determine the prevalence of hypovitaminosis D at HA and find the association of serum vitamin D levels with the risk factor for cardiovascular diseases. A cross‐sectional study of 350 Ladakhis and 50 lowlanders aged between 20 and 80 years was performed. The study population comprised different ethnic groups of Ladakh and lowlanders staying at HA. Anthropometric measures, blood pressure, pulse rate, complete blood count, liver function tests, kidney function tests, blood glucose, and oxygen saturation (SpO_2_) were measured. Coagulation and clinical parameters were analysed in plasma. The 25(OH) vitamin D was measured by competitive ELISA (DIAsource 25(OH) vitamin D Total ELISA). Of the study subjects, 9% (32) were obese, 28.7% (102) overweight, 53.8% (191) normal weight, and 8.45% (30) underweight. The mean SpO_2_ level in healthy Ladakhis was 89.90 ± 0.5986, and the mean SpO_2_ level in healthy lowlanders was 83.67 ± 1.265. The concentration of 25(OH) vitamin D in 130 subjects was insufficient (20–30 ng/ml), 175 were deficient (10–20 ng/ml), and 14 individuals had severe deficiency (<10 ng/ml) of vitamin D, whereas 66 had optimal levels (>30 ng/ml). Multiple linear regression analysis showed 25(OH) vitamin D association with various parameters such as age, BMI and Hb level. The study demonstrates a high prevalence of both 25(OH) vitamin D deficiency and insufficiency. The lowlanders staying at HA had a mean 25(OH) vitamin D level lower than the apparently healthy Ladakhis. This suggests susceptibility to various cardiovascular diseases associated with a reduced plasma level of vitamin D.

## ELUCIDATING INTERINDIVIDUAL VARIABILITY IN HYPOXIC RESPONSE AMONG HEALTHY INDIVIDUALS USING AYURGENOMICS APPROACH

Ritu Rani,^1,2,3^ Rintu Kutum,^2,3^ Anand Prakash Yadav,^1,4^ Anurag Agrawal,^5^ Mitali Mukerji,^1,2,6^ Vishal Bansal,^4^ Bhavana Prasher^1,2,3^

^1^Centre of Excellence for Applied Development of Ayurveda *Prakriti* and Genomics, CSIR‐Institute of Genomics and Integrative Biology, Delhi, India; ^2^CSIR's Ayurgenomics Unit—TRISUTRA (Translational Research and Innovative Science Through Ayurgenomics), CSIR‐Institute of Genomics and Integrative Biology, Mathura Road, New Delhi, India; ^3^Academy of Scientific and Innovative Research, Ghaziabad, Uttar Pradesh, India; ^4^Vallabhbhai Patel Chest Institute, University of Delhi, Delhi, India; ^5^Centre of Excellence for Translational Research in Asthma and Lung Disease, CSIR‐Institute of Genomics and Integrative Biology, Mall Road, Delhi, India; ^6^Indian Institute of Technology Jodhpur, NH 62, Karwar, Jodhpur, Rajasthan, India

The regulation of oxygen homeostasis is critical in physiology and disease pathogenesis. Physiological differences at baseline and magnitude and nature of responses to low oxygen levels or hypoxia vary considerably across individuals, which poses a significant challenge to predict outcomes. One way to stratify this variability could be an *Ayurvedic* approach that classifies healthy individuals into *Prakriti* types (*Vata*, *Pitta*, and *Kapha*) based on clinical phenotypes. Previously, we have shown genetic variations in *EGLN1* and *vWF* among healthy individuals of different Prakriti types that are associated with high‐altitude adaptation, susceptibility to HAPE, and thrombotic outcomes. To further elucidate variability in response to hypoxia, healthy individuals of different *Prakriti* were recruited and exposed to graded normobaric hypoxia. Physiological parameters such as heart rate (HR), heart rate variability (HRV), oxygen saturation (SpO_2_), and blood pressure (BP) were constantly monitored and the blood sample was collected before and after the hypoxia exposure for hematological and gene‐expression profile. HR was elevated and SpO_2_ and HRV were significantly reduced in an oxygen‐dependent manner. *Prakriti*‐specific effect on these parameters was also evident at baseline, as mean RR and systolic BP significantly varies between Prakriti groups. Additionally, we observed that *Kapha* individuals had a lower relative change (%Δ) in HR and HRV parameters than others, although not statistically significant. A further effect of hypoxia was assessed at the genome‐wide expression level in a subset of individuals. The analysis reveals that the protocol captures the immediate effects of hypoxic conditions found at high altitudes and also emphasizes the critical role of *Prakriti*‐based phenotyping in stratifying the differential responses to hypoxia.

## INVESTIGATING SINGLE AMINO ACID SUBSTITUTIONS IN ANGIOTENSINOGEN VIA STRUCTURAL GENOMIC APPROACHES

Bilal Ahmed Abbasi,^1^ Kavita Sharma^1^ Qadar Pasha^1,2^

^1^CSIR‐Institute of Genomics and Integrative Biology, Mall Road, Delhi, India; ^2^Institute of Hypoxia Research, New Delhi, India

Angiotensinogen (AGT), the precursor to angiotensin II, is a critical component of the renin–angiotensin system and plays a key role in vascular homeostasis. Mutations in AGT might induce abnormal structural changes and alter functionalities, which can lead to essential hypertension and other cardiovascular disorders. Herein, we have performed an extensive analysis of nonsynonymous single‐nucleotide polymorphisms resulting in single amino acid substitution in AGT to explore the role of disease‐causing variants and their structural dysfunctions. We used accessible database information and employed sequence‐based tools, structure‐based tools, and conservation analyses via in silico approaches. Several disruptive and pathogenic mutations have been identified based on the impact. Finally, two amino acid substitutions, rs699 (M268T) and rs4762 (T198M), were chosen for further investigation utilizing all‐atom molecular dynamics for 200 ns. Results indicate significant conformational altercations in the structure of AGT. Further, a case–control study with 517 controls and 524 patients confirmed the computational findings; the rs699C allele emerged significantly as the risk factor (odds ratio = 1.29; *p* = 3.8E − 03). Thus, this study provides substantial insight into the angiotensinogen dysfunction upon single amino acid alterations, which can be used to gain insights into the molecular basis of AGT‐associated disease progression and assist in therapeutics designing.

## OXADIAZINE DERIVATIVES AS PROMISING ANTIHEPATOTOXIC AGENTS: SYNTHESIS, BIOLOGICAL EVALUATION, AND MOLECULAR DYNAMICS INVESTIGATIONS

Saleem Akbar, Rikeshwer Prasad Dewangan, Bahar Ahmed

Anti‐hepatotoxic Research Laboratory, Department of Pharmaceutical Chemistry, SPER, Jamia Hamdard, New Delhi, India

Herbal treatments are widely popular for treating liver problems; however, they are still unacceptable due to a lack of herbal drug standardization. As a result, potential synthetic therapeutics are required to address the essential liver condition. In this study, we use the 1, 3, 4‐oxadiazine ring to find improved antihepatotoxic drugs by a suitable synthetic method. Analytical and spectral data were used to corroborate the structure of these oxadiazine‐based derivatives, which were then tested for antihepatotoxic potential. In vitro hepatotoxicity tests were also performed to determine the toxicity level of the developed compound; in vitro results led to the selection of compounds 5a, 5b, 5c, and 9d for further biological study. Afterward, in vivo antihepatotoxicity activity was assessed using a CCl4‐induced animal model. Compounds 5a and 5b, with 52.99%, 59.3%, 79.34% and 52.16%, 57.65%, 75.10%, respectively, were shown to be the most promising for lowering SGPT, SGOT, and ALKP levels. Furthermore, it was discovered that compound 5a had 411.01% albumin and 53.39% total protein, while compound 5b had 378.63% albumin and 48.9% total protein. The findings of induced‐fit docking for compounds 5a and 5b indicate some important binding information, as well as desirable ADME features and compliance with Lipinski's rule of five. Furthermore, 100‐ns molecular dynamics experiments demonstrate the protein–ligand complex's stability, corroborating in vitro and in vivo findings, and aid in determining the SAR of synthesized compounds. The antihepatotoxic activity of the two compounds, 5a and 5b, was higher than that of the conventional medication silymarin.

## REGULATION OF THROMBOSIS BY ALLEVIATING FIBROBLAST GROWTH FACTOR‐2 THROUGH INFLAMMASOME

Shankar Chanchal, Aastha Mishra, Swati Sharma, Raishal Safdar, Syed Mohd, Mohammad Zahid Ashraf

Department of Biotechnology, Jamia Millia Islamia, New Delhi, India

Hypoxia‐driven downstream signaling contributes to the regulation of thrombus formation. NLRP3 inflammasome has been reported to be directly associated with hypoxia‐inducible factor‐1α (HIF‐1α), which potentiates thrombosis under a hypoxic environment. Inflammation precedes coagulation coupled with a relative increase in the expression of NLRP3, caspase‐1, IL‐1β, and IL‐18 in thrombosis. Recent studies highlighted that fibroblast growth factor (FGF‐2) has a wide range of biological functions such as cellular proliferation, differentiation, and prolongation of endothelial cellular growth. The current study elucidated the role of FGF‐2 and HIF‐1α in thrombosis. To ascertain the level of messenger RNA expression of target genes under different conditions, THP‐1 cells were cultured and treated with hypoxia, DMOG (HIF‐1α activator), DIM (HIF‐1α inhibitor), and MCC950 (NLRP3 inhibitor). After treatment, RNA was extracted, and reverse transcription‐PCR was carried out for the expression level of target genes. The cell lysate was used for ELISA of the target protein. Results of DMOG treatment showed upregulation of NLRP3, caspase‐1, cytokines Il‐1β and factor III and downregulation of FGF‐2. THP1 cells treated with DMOG and hypoxia showed significantly decreased the expression of FGF‐2 in a concentration‐dependent manner as compared to control cells. Cells treated with DIM and MCC950 showed significantly increased FGF‐2 expression as compared to cells treated with DMOG. These observations revealed that the activity of FGF‐2 depends on the expression pattern of HIF‐1α and NLRP3, hence reflecting its involvement in thrombosis. In conclusion, FGF‐2 expression is modulated by HIF‐1α and NLRP3, which adds a new dimension to hypoxia‐induced thrombogenesis.

## EFFECTS OF ACUTE NORMOBARIC HYPOXIC EXPOSURE AND CAPNIA CONTROL ON MYOCARDIAL INOTROPISM ASSESSED BY KINOCARDIOGRAPHY

Cyril Tordeur,^1,2^ Jeremy Rabineau,^2^ Vitalie Faoro^1^

^1^Laboratory of Cardio‐Pulmonary Exercise Physiology Faculty of Motor Sciences, Université Libre de Bruxelles, Brussels, Belgium; ^2^Laboratory of Physics and Physiology—LPHYS Department of Cardiology, Erasme Hospital, Université Libre de Bruxelles, Brussels, Belgium

The increase in cardiac output resulting from acute hypoxic exposure is mainly explained by the increase in heart rate (HR), the stroke volume (SV) remaining unaffected. Kinocardiography is a technique based on the combination of ballistocardiography, reflecting hemodynamic activity, and seismocardiography, influenced by mechanical cardiac activity at each cardiac cycle. The objective of this randomized complete counterbalanced crossover study is to investigate the hemodynamic and cardiac mechanical effects of five exposure conditions: three levels (21%, 16%, 13%) of a fraction of inspired oxygen (F_i_O_2_) with and without control of capnia in normobaric hypoxia. Recordings were made on 16 healthy volunteers (26 ± 2 y), 60‐degree seated, during the first 5 min of exposure with a face mask. The volunteers were asked to stay still and not talk while watching the same movie; sufficient washout time was allowed between each condition. In accordance with previous work, SV, measured by finger continuous noninvasive blood pressure, did not change between the different conditions and HR increased with decreasing F_i_O_2_ (*p* < 0.001). Interestingly, the 13% F_i_O_2_ poïkilocapnic condition, compared to the normoxic condition (21% F_i_O_2_), induced a significant increase (adj. *p* = 0.027) in ${\text{iK}}_{\text{QT}}^{\text{SCG}}$, a surrogate for the myocardial contractility. In conclusion, the present results show that a stable SV needed to achieve an efficient oxygen transport in hypoxia requires, depending on the hypoxic stress level, an increase in myocardial contractility and ventricular ejection energy.

## ULTRASTRUCTURAL IMAGING AND QUANTIFICATION METHODS FOR IDENTIFYING THE EXTRACELLULAR VESICLES IN THE PULMONARY ARTERY BY ELECTRON MICROSCOPY

Shikha Chaudhary,^1^ Sushil Kumar,^2^ Subhash Chandra Yadav^1^

^1^Electron Microscope Facility, Anatomy, All India Institute of Medical Sciences, New Delhi, India; ^2^Cardiovascular Pulmonary Research Laboratories, Departments of Pediatrics and Medicine, School of Medicine, University of Colorado Anschutz Medical Campus, Aurora, Colorado, USA

Pulmonary hypertension (PH), a severe cardiopulmonary disease, was reported to be stimulated by extracellular vesicles (EVs) containing a complement system released by perivascular and adventitial fibroblast to activate the proinflammatory macrophages. Many researchers have provided indirect molecular‐level evidence for the activation of macrophages using flow cytometric approaches combined with RNA sequencing in hypoxic mouse models of PH. However, direct pieces of evidence of the release of these EVs from the fibroblast in the pulmonary artery were rarely reported. We have developed a method of sample preparation and electron microscope imaging techniques to directly identify these EVs in the multiple zones of pulmonary arteries taken from the calf under normal and hypoxic conditions in Karnovsky fixed samples. These samples were fixed and embedded to have the transverse section under the ultramicrotomy. The samples were primarily stained for optical microscopy to correlate and identify the different zone of the pulmonary artery. Each location was magnified sequentially to finally observe the EVs in each site of pulmonary arteries. We have observed many fibroblasts where these EVs were under synthesis, releasing, and in the released condition in the collagen fiber‐rich tissue. We have also developed a quantification technique based on transmission electron microscope imaging. Using this indigenous developed protocol, we have observed the highest number of EVs in the intima zone, followed by media, and least in the adventitia zone. This preliminary study will help to directly validate the mechanism of macrophage activation through these EVs in PH.

## MODELING CHROMATINOPATHIES IN A DISH: A REPRESENTATIVE EXAMPLE USING *EHMT1* GENE

Radhika Rao Arsala,^1^ Pooja Shukla,^2^ Kriti K. B.^2^ Shravanti Rampalli^2^

^1^Institute for Stem Cells and Regenerative Medicine, Bengaluru, Karnataka, India; ^2^Cardiorespiratory Disease Biology, CSIR‐Institute of Genomics and Integrative Biology, New Delhi, India

Chromatinopathies are complex, heritable developmental disorders that manifest in early childhood and are characterized by abnormalities in cognitive functions, verbal and nonverbal communication skills, learning and memory, and speech development. For example, mutations in chromatin modulators such as *MeCP2*, *NSD1*, *ATXN1*, *JARID2*, and *EHMT1* are responsible for the manifestation of chromatinopathies. Euchromatic histone methyltransferase 1 (EHMT1)/GLP (HMT family) is a ubiquitously expressed chromatin modifier, which is crucial for mono/dimethylation of lysine 9 of histone 3 in euchromatic regions and leads to silencing of targeted genes. It has been reported that haploinsufficiency (due to mutation or deletion) of euchromatin *EHMT1* leads to 9q34.3 subtelomeric deletion syndrome or Kleefstra syndrome (KS). The KS patients show severe intellectual disability with respect to learning and memory and other physical symptoms like hypotonia, microcephaly, brachycephaly, hypertelorism, anteverted nares, macroglossia, obesity, and congenital heart defects. The *EHMT1* plays an important role in mouse development as its knockout leads to embryonic lethality. Additionally, it has been implicated in germ cell, B‐ and T‐cell development and brown fat specification. At the cellular level, loss of *EHMT1* contributes to reduced branching and spine density of hippocampal neurons and synaptic dysfunctions in the mouse model. While the above studies indicate the crucial role of *EHMT1*, its mechanistic contributions to neural development and pathogenesis remain elusive. In the current study, we engineered mutant human pluripotent cell lines lacking or haploinsufficient for *EHMT1* and study the differentiation potential of neural development.

